# Medin co-aggregates with vascular amyloid-β in Alzheimer’s disease

**DOI:** 10.1038/s41586-022-05440-3

**Published:** 2022-11-16

**Authors:** Jessica Wagner, Karoline Degenhardt, Marleen Veit, Nikolaos Louros, Katerina Konstantoulea, Angelos Skodras, Katleen Wild, Ping Liu, Ulrike Obermüller, Vikas Bansal, Anupriya Dalmia, Lisa M. Häsler, Marius Lambert, Matthias De Vleeschouwer, Hannah A. Davies, Jillian Madine, Deborah Kronenberg-Versteeg, Regina Feederle, Domenico Del Turco, K. Peter R. Nilsson, Tammaryn Lashley, Thomas Deller, Marla Gearing, Lary C. Walker, Peter Heutink, Frederic Rousseau, Joost Schymkowitz, Mathias Jucker, Jonas J. Neher

**Affiliations:** 1 German Center for Neurodegenerative Diseases (DZNE), Tübingen, Germany; 2 Department of Cellular Neurology, Hertie Institute for Clinical Brain Research, University of Tübingen, Tübingen, Germany; 3 Graduate School of Cellular and Molecular Neuroscience, University of Tübingen, Tübingen, Germany; 4 Switch Laboratory, VIB-KU Leuven Center for Brain and Disease Research, Leuven, Belgium; 5 Switch Laboratory, Department of Cellular and Molecular Medicine, KU Leuven, Leuven, Belgium; 6 Department of Cardiovascular and Metabolic Medicine, Institute of Life Course and Medical Sciences, University of Liverpool, Liverpool, UK; 7 Liverpool Centre for Cardiovascular Sciences, University of Liverpool, Liverpool, UK; 8 Department of Biochemistry and Systems Biology, Institute of Systems, Molecular and Integrative Biology, University of Liverpool, Liverpool, UK; 9 Monoclonal Antibody Core Facility, Institute for Diabetes and Obesity, Helmholtz Zentrum München, Research Center for Environmental Health, Neuherberg, Germany; 10 German Center for Neurodegenerative Diseases (DZNE), Munich, Germany; 11 Institute of Clinical Neuroanatomy, Dr. Senckenberg Anatomy, Neuroscience Center, Goethe University, Frankfurt/Main, Germany; 12 Department of Physics, Chemistry and Biology, Linköping University, Linköping, Sweden; 13 Queen Square Brain Bank for Neurological Disorders, University College London Queen Square Institute of Neurology, London, UK; 14 Department of Neurodegenerative Disease, University College London Queen Square Institute of Neurology, London, UK; 15 Department of Pathology and Laboratory Medicine and Department of Neurology, Emory University School of Medicine, Atlanta, GA USA; 16 Department of Neurology and Emory National Primate Research Center, Emory University, Atlanta, GA USA

**Keywords:** Alzheimer's disease, Alzheimer's disease, Alzheimer's disease, Neurodegeneration

## Abstract

Aggregates of medin amyloid (a fragment of the protein MFG-E8, also known as lactadherin) are found in the vasculature of almost all humans over 50 years of age^1,2^, making it the most common amyloid currently known. We recently reported that medin also aggregates in blood vessels of ageing wild-type mice, causing cerebrovascular dysfunction^3^. Here we demonstrate in amyloid-β precursor protein (APP) transgenic mice and in patients with Alzheimer’s disease that medin co-localizes with vascular amyloid-β deposits, and that in mice, medin deficiency reduces vascular amyloid-β deposition by half. Moreover, in both the mouse and human brain, MFG-E8 is highly enriched in the vasculature and both MFG-E8 and medin levels increase with the severity of vascular amyloid-β burden. Additionally, analysing data from 566 individuals in the ROSMAP cohort, we find that patients with Alzheimer’s disease have higher *MFGE8* expression levels, which are attributable to vascular cells and are associated with increased measures of cognitive decline, independent of plaque and tau pathology. Mechanistically, we demonstrate that medin interacts directly with amyloid-β to promote its aggregation, as medin forms heterologous fibrils with amyloid-β, affects amyloid-β fibril structure, and cross-seeds amyloid-β aggregation both in vitro and in vivo. Thus, medin could be a therapeutic target for prevention of vascular damage and cognitive decline resulting from amyloid-β deposition in the blood vessels of the brain.

## Abstract (web summary)

Medin promotes the formation of vascular aggregates with amyloid-β in mouse models and in human patients with Alzheimer’s disease, and is associated with vascular defects and cognitive decline.

## Main

Amyloidosis is caused by the local or systemic accumulation of insoluble, misfolded and aggregated proteins. Thirty-six amyloids have been identified so far, many of them associated with tissue dysfunction and disease^4^, including major neurodegenerative diseases^5,6^. The most common human amyloid known to date is medin, a 50-amino-acid peptide cleaved (by unknown mechanisms) from the protein MFG-E8 (milk fat globule EGF-like factor-8). Medin amyloid was first described in the aorta but is also found in other arteries of the upper body in around 97% of people of European descent above 50 years of age^1,2,7^. Previous studies have implied that medin aggregates may weaken and lead to the degeneration of the arterial wall, and may cause arterial stiffening and cerebrovascular dysfunction^8–12^. Recently, we performed a mechanistic study that showed that medin is also present in ageing wild-type mice, where it forms vascular aggregates that cause cerebrovascular dysfunction^3^. Independently, a recent analysis of postmortem human samples found that medin aggregates are increased in cerebral arterioles of patients with vascular dementia or Alzheimer’s disease compared with cognitively healthy controls, and that among cerebrovascular pathologies, arteriolar medin was the best predictor of Alzheimer’s disease diagnosis^9^. These findings raise the question of whether increased medin levels are a cause or a consequence of Alzheimer’s disease pathology. Therefore, we here study the role of medin in mouse models of cerebral β-amyloidosis and in postmortem human brain tissue.

## Medin promotes vascular amyloid-β deposition

**Fig. 1:**
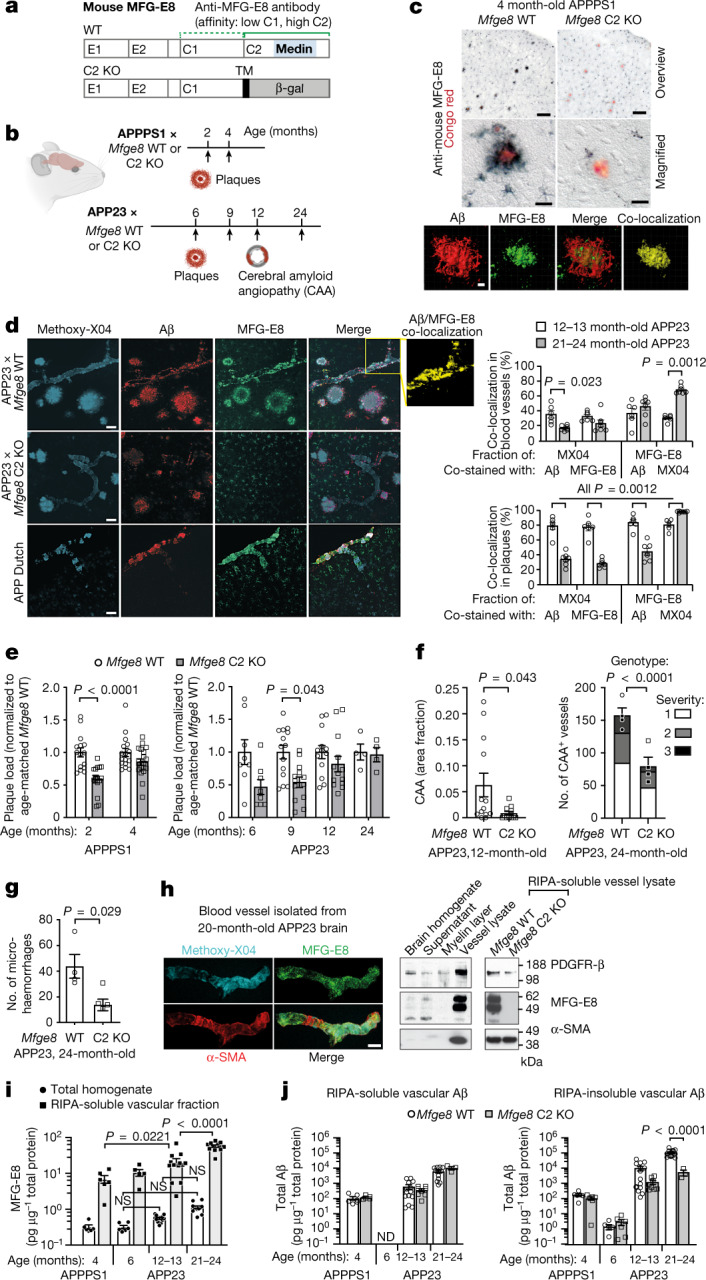
Medin co-localizes with amyloid-β deposits and promotes vascular β-amyloidosis in mouse models. **a**, Schematic of MFG-E8 protein domains in *Mfge8* wild-type (WT) and C2 domain knockout (C2 KO) mice and the binding sites of the anti-mouse MFG-E8 antibody (green; dotted and solid lines indicate weak and strong antibody affinity, respectively). TM, transmembrane domain; β-gal, β-galactosidase reporter gene . **b**, Schematic of pathology and timing of analyses (arrows) in APP transgenic lines. **c**,**d**, Immunostaining of cortical brain sections of 4-month-old APPPS1 × *Mfge8* wild-type or APPPS1 × *Mfge8* C2 KO mice (**c**) and 27-month-old APP Dutch mice (*n* = 2 females analysed) and 24-month-old APP23 mice (**d**, left), with quantification of co-localization (3 female and 3 male 12- to 13-month-old mice, and 2 female and 5 male 21- to 24-month-old mice; total of *n* = 323 vessels and *n* = 386 plaques). **c**, Bottom, reconstructed confocal *z*-stack. Aβ, amyloid-β. **e**, Plaque load in *Mfge8* wild-type and C2 KO mice in APPPS1 (2-month-old: 7 female and 8 male wild-type mice, 6 female and 9 male C2 KO mice; 4-month-old: 8 female and 8 male wild-type mice, 12 female and 8 male C2 KO mice) and APP23 lines (6-month-old: 8 female wild-type and 8 female C2 KO mice, males have no plaques yet; 9-month-old: 7 female and 7 male wild-type mice, 7 female and 6 male C2 KO mice; 12-month-old: 4 female and 9 male wild-type mice, 6 female and 7 male C2 KO mice; 24-month-old: 4 male wild-type mice, 4 male C2 KO mice). **f**,**g**, CAA-laden vessels in 12- and 24-month-old APP23 animals (**f**) and microhaemorrhages in 24-month-old APP23 animals (**g**) (numbers of mice as in **e**). **h**, Confocal *z*-stack of an isolated cerebral blood vessel and western blotting for vascular markers (α-smooth muscle actin, α-SMA; platelet-derived growth factor receptor-β, PDGFR-β) and MFG-E8. **i**, Quantification of MFG-E8 by ELISA (4-month-old APPPS1: 3 female and 3 male mice; 6-month-old APP23: 3 female and 3 male mice; 12- to 13-month-old APP23: 7 female and 7 male mice; 21- to 24-month-old APP23: 2 female and 10 male mice). **j**, Quantification of total vascular amyloid-β by ELISA (*Mfge8* wild-type as in **i**; APPPS1 × *Mfge8* C2 KO: 2 female and 3 male mice; 6-month-old APP23 × *Mfge8* C2 KO: 3 female and 3 male mice; 12- to 13-month-old APP23 × *Mfge8* C2 KO: 4 female and 4 male mice; 21- to 24-month-old APP23 × *Mfge8* C2 KO: 3 male mice). Data are mean ± s.e.m. **e**,**f**, right, **i**,**j**, Two-way-ANOVA with Tukey’s post hoc comparison. **d**,**f**, left, **g**, Two-tailed Mann–Whitney *U*-test. Scale bars: 100 µm (**c**, overview), 20 µm (**c**, magnified, **h**), 5 µm (**c**, *z*-stack) and 50 µm (**d**). ND, not detectable; NS, not significant. Uncropped western blots are shown in Supplementary Fig. 1. Source data

**Extended Data Fig. 1:**
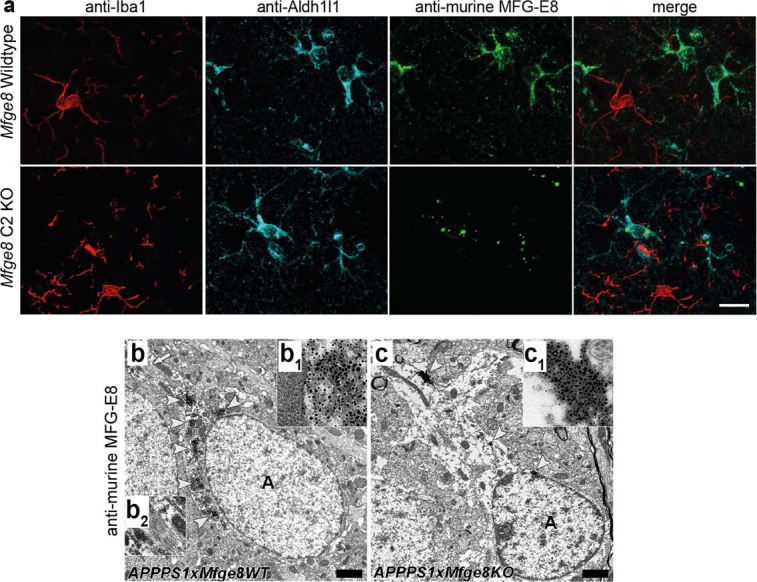
MFG-E8 is expressed primarily by astrocytes in APP transgenic mice. **a**, Immunofluorescent co-staining of MFG-E8 (green) with astrocytic (Aldh1l1, cyan) and microglial (Iba1, red) markers demonstrates co-localisation only with astrocytes. Note that in *Mfge8* C2 KO mice, the truncated MFG-E8/β-galactosidase fusion protein (cf. Fig. 1a) is still expressed but is retained inside the cell due to an artificial transmembrane domain, causing a punctate staining pattern. **b**, Astrocytic (‘A’) staining is also evident by immuno-electron microscopy in 4-month-old APPPS1 x *Mfge8* wild-type (WT) animals (arrowheads indicate positively stained structures, which are magnified in inset b_1_). The arrow *(top left)* indicates bundles of intermediate filaments in the astrocytic cytoplasm, which are shown at higher magnification in b_2_. **c**, Astrocytes in *Mfge8* C2 KO animals also contain heavily labelled punctate structures, indicating intracellular accumulation of the truncated MFG-E8 protein (arrowheads indicate positively stained structures, which are magnified in c_1_ inserts; n = 1f/1m *Mfge8* WT and n = 1f/1m *Mfge8* C2 KO animals analysed). Scale bar; 15 µm in *a*, 500 nm in *b*.

First, we stained brain tissue from two APP transgenic mouse lines, APPPS1 and APP23^13,14^, using a polyclonal anti-mouse MFG-E8 antibody. We previously established that this antibody recognizes extracellular medin aggregates in the vasculature of ageing wild-type mice owing to its high affinity for the medin-containing C2 domain, but it also detects the C1 domain with lower affinity^3^ (Fig. 1a). Staining with this antibody co-localized extensively with amyloid plaques in both mouse models (Figs. 1c,d). Accordingly, when we genetically eliminated medin by cross-breeding the APP transgenic lines with mice lacking the medin-containing C2 domain of *Mfge8*^15^ (*Mfge8* C2 KO; Fig. 1a,b), amyloid plaque staining was entirely absent. However, punctate staining was still visible in astrocytes owing to the intracellular retention of the truncated MFG-E8 C2 KO variant in *Mfge8*-expressing cells (Figs. 1a,c,d and Extended Data Fig. 1). In addition to plaque staining, and in line with the reported vascular localization of medin amyloid^7,9^, we also noted co-localization of MFG-E8 staining with cerebral β-amyloid angiopathy (CAA)—that is, vascular amyloid-β deposition. Vascular MFG-E8 staining was conspicuous both in APP23 mice (which develop CAA starting from around 12 months of age^14,16^) and in a second CAA model, APP Dutch mice^17^ (Fig. 1d) but was absent in APPPS1 mice, which do not develop CAA to any robust degree^13^, and was also eliminated in APP23 mice crossed with the medin-deficient *Mfge8* C2 KO line (Fig. 1d).

To examine the association of MFG-E8 and medin with amyloid-β deposits in more detail, we performed 3D reconstructions of confocal *z*-stacks from brain sections of APP23 mice (as they show both plaque and CAA pathology) stained for MFG-E8 (or fragments thereof), amyloid-β and the amyloid-binding dye Methoxy-X04. In plaques, around 81% of Methoxy-X04 staining co-localized with amyloid-β in 12- to 13-month-old APP23 mice but this fraction decreased to about 34% in 21- to 24-month-old mice, possibly owing to reduced antibody penetration into highly compact amyloid aggregates in aged mice^18^. This was similar for MFG-E8 staining, with around 79% and 28% of Methoxy-X04 co-staining with MFG-E8 fragments in the respective age groups. Performing the inverse analysis—that is, examining the fraction of MFG-E8 co-staining with Methoxy-X04 or amyloid-β—we found that the vast majority of MFG-E8 staining in plaques co-localized with Methoxy-X04 staining—specifically, 81% in 12- to 13-month-old mice and 99% in 21- to 24-month-old mice. Thus, nearly all MFG-E8 (or fragments) was directly associated with amyloid fibrils in plaques of aged mice. By contrast, when quantifying the co-localization of amyloid-β with MFG-E8 antibody staining in plaques, we found that the co-localization of MFG-E8 fragments with amyloid-β decreased notably from around 84 to 44% with increasing age. This indicated that at later stages of plaque pathology, a smaller amount of MFG-E8 (or fragments) may be interacting with less compact (Methoxy-X04-negative) forms of amyloid-β (Fig. 1d). Focussing on CAA next, we found that a smaller fraction of Methoxy-X04 staining co-localized with amyloid-β and MFG-E8 (or fragments), 36% and 17% for amyloid-β staining, and 33% and 23% for MFG-E8 staining in 12- to 13-month-old and 21- to 24-month-old APP23 mice, respectively. Similar to plaques, the fraction of MFG-E8 staining co-localizing with Methoxy-X04 staining in blood vessels increased from 31% to 70% in 12- to 13-month-old and 21- to 24-month-old APP23 mice, respectively. However, in contrast to plaques, the proportion of MFG-E8 staining co-localizing with amyloid-β in blood vessels slightly increased (albeit non-significantly) from 37% to 46% with age in APP23 mice (Fig. 1d). This suggested that in the vasculature, MFG-E8 (or fragments) interact with less compact (Methoxy-X04-negative) as well as compact amyloid-β aggregates, even at advanced stages of CAA pathology.

**Extended Data Fig. 2:**
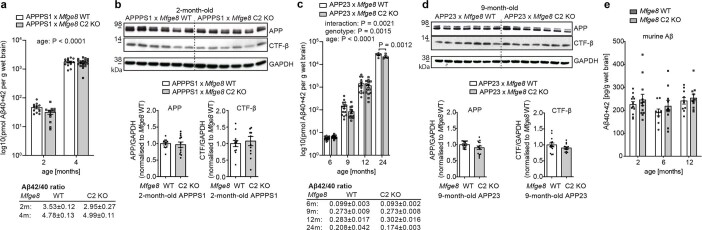
MFG-E8/medin deficiency does not strongly affect Aβ generation and Aβ protein levels. **a**/**b**, Biochemical analysis of Aβ levels and APP levels/processing in APPPS1 x *Mfge8* WT/C2 KO animals (2 months: n = 6f/6m APPPS1 x *Mfge8* WT and n = 6f/6m *Mfge8* C2 KO, 4 months n = 6f/8m WT and n = 12f/8m C2 KO). **a**, ELISA measurements of Aβ40 and Aβ42 levels in brain homogenate of APPPS1 x *Mfge8* WT/C2 KO animals. **b**, Quantification of Amyloid Precursor Protein (APP) and its C-terminal fragment-β (CTF-β) by Western Blotting analysis (using 6E10 antibody) in brain homogenates from 2-month-old APPPS1 x *Mfge8* WT/C2 KO animals (which show the most pronounced changes in Aβ levels); data were pooled from different blots after normalisation to the average wild-type level, with a representative blot shown. **c**/**d**, Biochemical analysis of Aβ levels and APP levels/processing in APP23 x *Mfge8* WT/C2 KO animals (6 months n = 8f/6m WT and n = 8f/6m *Mfge8* C2 KO, 9 months n = 7f/7m WT and n = 7f/6m C2 KO; 12 months n = 7f/7m WT and n = 7f/7m *Mfge8* C2 KO; 24 months: n = 5m WT and n = 4m C2 KO). **c**, ELISA measurements of Aβ40 and Aβ42 levels in brain homogenate of APP23 x *Mfge8* WT vs. APP23 x *Mfge8* C2 KO animals. **d**, Quantification of APP and CTF-β by Western Blotting analysis (using 6E10 antibody) in brain homogenates from 9-month-old APP23 x *Mfge8* WT/C2 KO animals (which show the most pronounced changes in Aβ levels in the absence of CAA); data were pooled from different blots after normalisation to the average wild-type level, with a representative blot shown. **e**, ELISA measurements of murine Aβ40 and Aβ42 levels in (APP non-transgenic) *Mfge8* WT vs. *Mfge8* C2 KO animals (2 months: n = 6m/6f *Mfge8* WT and n = 6m/6f *Mfge8* C2 KO, 6 months n = 4m/5f *Mfge8* WT and n = 4m/6f *Mfge8* C2 KO; 12 months n = 4m/6f *Mfge8* WT and n = 3m/7f *Mfge8* C2 KO). Data shown are means±S.E.M and were analysed by 2-way ANOVA, followed by Tukey’s posthoc comparisons. Uncropped Western Blots are shown in Supplementary Fig. 4. Source data

**Extended Data Fig. 3:**
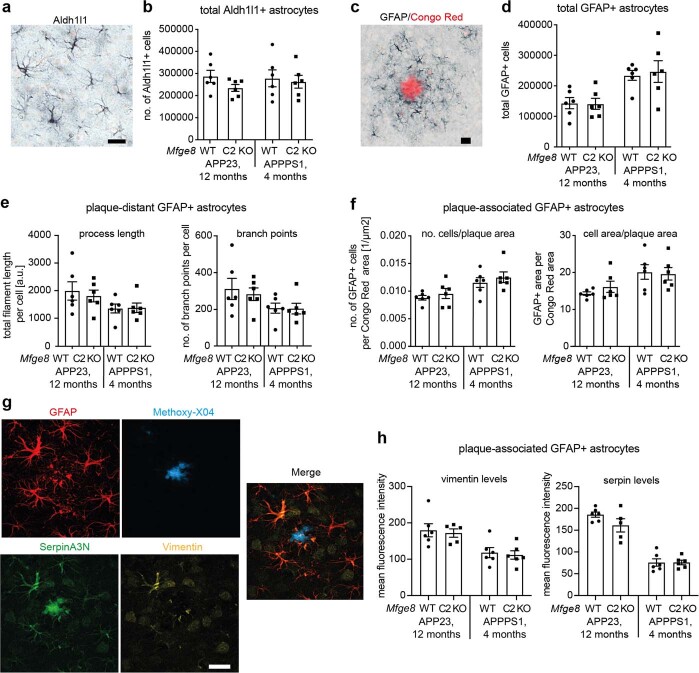
MFG-E8/medin deficiency does not affect astrocytic responses to cerebral β-amyloidosis. Astrocytic responses to cerebral β-amyloidosis were assessed in 12-month-old APP23 and 4-month-old APPPS1 x *Mfge8* WT/C2 KO animals (n = 3m/3f per line and genotype for all analyses), because in these age groups, plaque load is indistinguishable between *Mfge8* WT/C2 KO animals, allowing for a direct comparison between genotypes. None of the measures showed genotype-dependent effects. **a**, Homeostatic astrocytes were stained for Aldh1l1 and **b**, quantified stereologically. **c**, Activated astrocytes were stained for GFAP in combination with Congo Red (to label amyloid plaques), and **d**, stereologically quantified. **e**, The morphology of astrocytes was examined by quantifying total process length and number of branch points in reconstructed confocal z-stacks based on GFAP staining (total number of individual cells analysed: APP23: 33/37 in *Mfge8* WT/C2 KO; APPPS1: 67/66 in *Mfge8* WT/C2 KO animals). **f**, Plaque-associated astrocytes were quantified stereologically in a fixed radius around amyloid plaques. **g**, Astrocyte activation was further assessed using two recently reported markers of “disease-associated astrocytes”, serpinA3N and vimentin^68^. **h**, Quantification of mean fluorescence intensities of vimentin and serpinA3N in GFAP-positive astrocytes. Data shown are means per animal ± S.E.M. Scale bar: 25 µm in *a*/*c*/*g*. Source data

**Extended Data Fig. 4:**
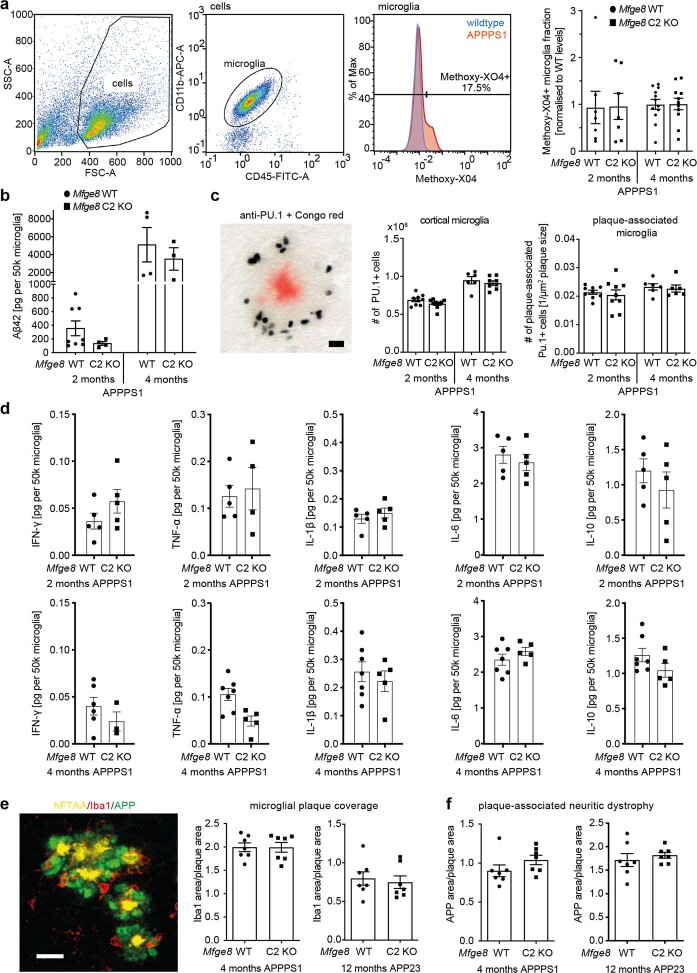
MFG-E8/medin deficiency does not affect microglial responses to cerebral β-amyloidosis. **a**, Microglial phagocytosis of aggregated Aβ is not affected by *Mfge8* C2 KO in APPPS1 animals, as evidenced by FACS-based analysis of the amyloid-specific Methoxy-X04 signal (2 months: n = 4f/4m *Mfge8* WT n = 5f/2m *Mfge8* C2 KO; 4 months: n = 9f/3m *Mfge8* WT, n = 6f/5m *Mfge8* C2 KO) in microglia (identified based on CD11b^high^, CD45^intermediate^ cells) or by **b**, Ultra-sensitive ELISA measurements of Aβ42 levels in purified microglia (as in *a*) (2 months APPPS1: n = 5f/3m *Mfge8* WT, n = 3f/2m *Mfge8* C2 KO; 4 months APPPS1: n = 1f/3m *Mfge8* WT, n = 2f/1m *Mfge8* C2 KO). **c**, Stereological quantification of total cortical microglia (PU.1 staining, black) or plaque-associated microglia (plaques stained with Congo Red) shows no differences in total and plaque-associated numbers of microglia in APPPS1 x *Mfge8* WT versus APPPS1 x *Mfge8* C2 KO animals at 2/4 months of age (for total microglia in 2-month-old animals: n = 5f/4m APPPS1 x *Mfge8* WT and n = 6f/3m APPPS1 x *Mfge8* C2 KO and in 4-month-old animals: n = 3f/3m APPPS1 x *Mfge8* WT and 6f/2m APPPS1 x *Mfge8* C2 KO; for plaque-associated microglia in 2-month-old animals: n = 5f/4m APPPS1 x *Mfge8* WT and n = 6f/3m APPPS1 x *Mfge8* C2 KO; in 4-month-old animals: 5f/1m APPPS1 x *Mfge8* WT and 3f/3m APPPS1 x *Mfge8* C2 KO). **d**, Cytokine levels measured in FACS-purified microglia are indistinguishable in 2- and 4-month-old APPPS1 x *Mfge8* WT and APPPS1 x *Mfge8* C2 KO animals APPPS1 (2 months: n = 5f/3m *Mfge8* WT, n = 3f/2m *Mfge8* C2 KO; 4 months: n = 3f/4m *Mfge8* WT, n = 3f/2m *Mfge8* C2 KO). **e**/**f**, Using staining for amyloid (hFTAA), microglia (Iba1) and dystrophic neurites (APP), the microglial plaque barrier (*e*) and plaque-associated neuritic dystrophy (*f*) were quantified in 12-month-old APP23 and 4-month-old APPPS1 x *Mfge8* WT/C2 KO animals, because in these age groups, plaque load is indistinguishable between *Mfge8* WT/C2 KO animals (n = 3f/4m analysed for each group), allowing for a direct comparison between genotypes. Neither measure was significantly altered in *Mfge8* WT vs. C2 KO animals. Data shown are means ± S.E.M. Scale bar: 20 µm in c/e. Source data

Having established substantial co-localization of MFG-E8 and/or its fragments with amyloid-β deposits, we next examined the role of MFG-E8 and medin in β-amyloidosis by quantifying plaque and CAA load in the APPPS1 × *Mfge8* wild-type or C2 KO and APP23 × *Mfge8* wild-type and C2 KO mice. Notably, the lack of extracellular MFG-E8 or medin in *Mfge8* C2 KO mice significantly reduced plaque deposition at early stages of pathology in both APP lines. In particular, amyloid-β plaque load was reduced by around 40% in 2-month-old APPPS1 × *Mfge8* C2 KO mice compared with APPPS1 × *Mfge8* wild-type mice (Fig. 1e) (with APPPS1 mice showing first plaques around 6 weeks) and by around 50% in 6- and 9-month-old APP23 × *Mfge8* C2 KO mice compared with APP23 × *Mfge8* wild-type mice (Fig. 1e) (with APP23 mice showing first plaques around 6 months of age). However, in both models, parenchymal plaque load was indistinguishable at later stages of amyloid-β pathology (Fig. 1e), in line with our observation that a smaller amount of MFG-E8 (or fragments) may be integrated into amyloid-β aggregates at later stages of disease (Fig. 1d). Notably, in brain homogenates, total amyloid-β measurements showed only tendencies towards reduced levels in APP × *Mfge8* C2 KO mice (measured by enzyme-linked immunosorbent assay (ELISA) in formic acid extracts; Extended Data Fig. 2), indicating that the lack of MFG-E8 or medin did not affect global amyloid-β levels. Accordingly, protein levels and processing of transgenic human APP were unchanged between *Mfge8* genotypes in both APP transgenic mouse lines. Similarly, levels of mouse amyloid-β in APP non-transgenic *Mfge8* wild-type and *Mfge8* C2 KO mice were indistinguishable (Extended Data Fig. 2). To examine whether the absence of functional MFG-E8 may affect astrocytic or microglial responses, we also quantified the numbers of these cells, astrocyte morphology and activation markers, microglial plaque coverage and amyloid-β phagocytosis as well as cytokine levels. Again, we did not detect any effects of the *Mfge8* genotype on these glial cells in either of the two APP mouse models (Extended Data Figs. 3 and 4).

Next, we examined the effect of MFG-E8 and/or medin on CAA burden in APP23 mice. Despite the high variability in CAA levels in 12-month-old APP23 mice, *Mfge8* C2 KO mice showed a reduction of around 85% in CAA burden compared with *Mfge8* wild-type mice (Fig. 1f). Moreover, in 24-month-old APP23 mice, which show severe CAA that results in microhaemorrhages^19^, the CAA burden and the number of microhaemorrhages were reduced by about 50% and 65%, respectively, in *Mfge8* C2 KO mice compared with *Mfge8* wild-type mice (Fig. 1f,g). These data indicate that MFG-E8 and/or medin promote amyloid-β aggregation, particularly in the vasculature, in line with our co-localization analysis suggesting a continuous integration of MFG-E8 and/or medin in vascular amyloid deposits (Fig. 1d).

**Extended Data Fig. 5:**
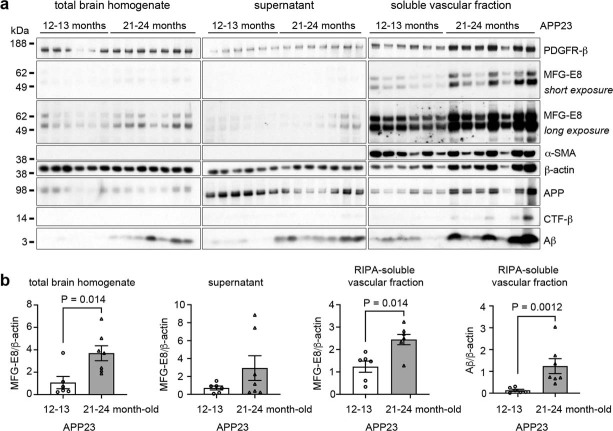
MFG-E8 levels increase with age and cerebral β-amyloidosis in APP23 mice. Validation of ELISA results presented in Fig. 2 using **a**, Western blotting of total mouse brain homogenates, brain homogenate ‘supernatant’, and isolated blood vessels of adult (12–13-month-old) and aged (21–24-month-old) APP23 mice (see Methods for details of the isolation protocol). With increasing Aβ levels, APP23 mice show higher MFG-E8 levels in the soluble vascular fraction, confirming ELISA measurements (cf. Fig. 1i). PDGFR-β and α-SMA were used as vascular markers, β-actin was used as the loading control. **b**, Quantification of Western Blot bands (adult: n = 3f/3m, aged: n = 2f/5m, two-tailed Mann-Whitney test). Blots were repeated at least two times, with equivalent results. Data shown are means ± S.E.M. Uncropped Western Blots are shown in Supplementary Fig. 5. Source data

Next, to assess why medin may affect amyloid-β aggregation specifically at the vascular level, we isolated cerebral blood vessels from our mouse models (Fig. 1h). In cerebral blood vessels from APPPS1 and APP23 mice, MFG-E8 protein levels were strongly enriched in soluble vascular fractions compared with total brain homogenates from the same mice (approximately 20-fold enrichment in 4-month-old APPPS1; and approximately 35-, 38- and 53-fold enrichment in 6-, 12- to 13-, and 21- to 24-month-old APP23 mice, respectively). Moreover, MFG-E8 levels were further increased with CAA pathology; in particular, despite similar total amyloid-β levels in the brain (Extended Data Fig. 2), MFG-E8 levels were about threefold higher in 12- to 13-month-old APP23 mice (with mild CAA) compared with 4-month-old APPPS1 mice (without CAA). MFG-E8 levels were also increased in an age- and pathology-dependent manner in APP23 mice, increasing by around twofold in 12- to 13-month-old mice and by more than fivefold in 21- to 24-month-old mice compared with 6-month-old mice with no CAA pathology (measured by ELISA (Fig. 1i) and validated by western blotting, Extended Data Fig. 5).

**Extended Data Fig. 6:**
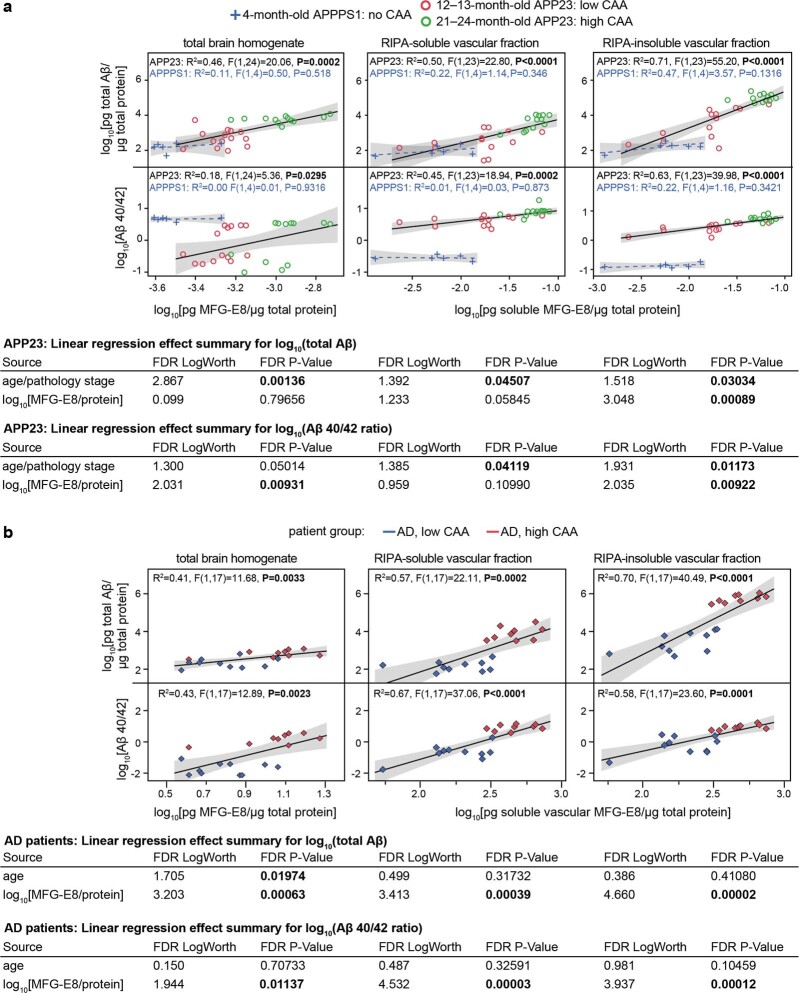
MFG-E8 levels correlate with vascular Aβ levels in mice and AD patients. MFG-E8 and Aβ38/40/42 were measured by ELISA in total brain homogenate (left panels), and in RIPA-soluble (middle panels) and insoluble fractions (right panels) of isolated cerebral blood vessels from mouse (*a*; n as in Fig. 1i) and human brain tissue (*b*; n as in Fig. 2e). As MFG-E8 ELISA measurements were not possible in the formic acid-extracted insoluble vascular fraction, correlations were performed for MFG-E8 levels in the soluble vascular fraction. **a**, Correlations of MFG-E8 with total Aβ levels (Aβ38+Aβ40+Aβ42) as well as the Aβ40/42 ratio in brain tissue from APPPS1 mice without CAA versus 12–13- and 21–24-month-old APP23 mice with low and high CAA, respectively. *Bottom*, Linear regression analysis was performed considering the age/pathology-stage of APP23 animals as an independent factor (analyses are shown underneath their respective graphs). While this reduces or even eliminates the predictive effects of MFG-E8 levels on Aβ levels for some comparisons, effects remain highly significant for the RIPA-insoluble vascular fraction, confirming the role of MFG-E8/medin in driving vascular Aβ aggregation. **b**, Correlations of MFG-E8 with total Aβ levels as well as the Aβ40/42 ratio in brain tissue from AD patients with low and high levels of CAA (as we could not reliably measure Aβ in control patients, these are not included). *Bottom*, Linear regression analysis with age as an independent factor does not eliminate the predictive effects of MFG-E8 levels on Aβ levels in human samples. FDR: False Discovery Rate (adjusted P-value using the Benjamini-Hochberg correction). FDR LogWorth: -log10(FDR P-Value) for each model effect. Shaded areas indicate 95% confidence intervals. Source data

To corroborate our histological finding that the lack of medin reduces CAA, we also measured total amyloid-β levels in isolated cerebral blood vessels (Fig. 1j). Amyloid-β levels in the soluble vascular fraction did not differ between APP × *Mfge8* wild-type and C2 KO mice. However, insoluble vascular amyloid-β (measured after formic acid extraction) was around eightfold lower (albeit not significantly, owing to the high variation in CAA in the *Mfge8* wild-type group) in 12- to 13-month-old APP23 × *Mfge8* C2 KO mice and reduced (highly significantly) by around 20-fold in 21- to 24-month-old APP23 × *Mfge8* C2 KO mice compared with wild-type mice. By contrast, levels of soluble and insoluble vascular amyloid-β were unaffected by *Mfge8* genotype in 6-month-old APP23 mice and 4-month-old APPPS1 mice without CAA pathology (Fig. 1j). To further validate these findings, we also examined the relationship between MFG-E8 levels and the Aβ40/Aβ42 ratio (an indicator of CAA^20,21^) as well as amyloid-β levels in total brain homogenate and soluble and insoluble vascular fractions from APP × *Mfge8* wild-type mice. In APP23 mice, but not in APPPS1 mice, MFG-E8 levels correlated positively with both the Aβ40/Aβ42 ratio and total amyloid-β levels. Notably, these correlations were strongest for the insoluble vascular fraction and weakest for total brain homogenate (Extended Data Fig. 6), corroborating the association of increased MFG-E8 levels specifically with CAA in mice (as also indicated by a recent independent study^22^). Although we could not confirm the presence of medin in our mouse samples biochemically (owing to the lack of a high affinity antibody for western blotting of mouse medin), these data demonstrate that the medin precursor protein MFG-E8 is highly enriched in the vasculature and is further increased with CAA, providing an explanation for why genetic medin deficiency may primarily affect vascular amyloid-β aggregation.

## Medin increases with CAA in Alzheimer’s disease

**Fig. 2:**
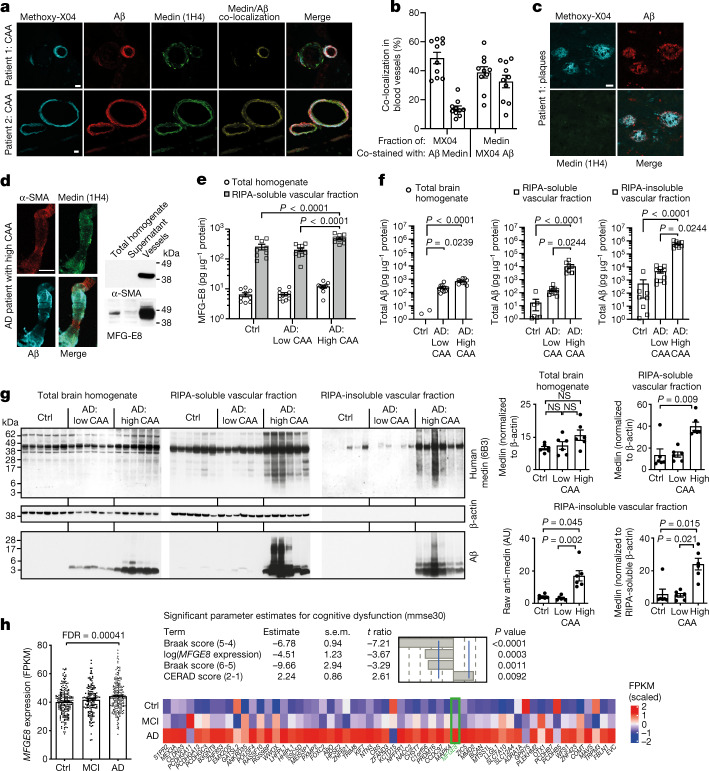
MFG-E8 is highly enriched in the human brain vasculature, increases with CAA and is associated with cognitive dysfunction in patients with Alzheimer’s disease. **a**–**c**, Analysis of human brain sections from patients with Alzheimer’s disease (*n* = 16 patients analysed), stained for medin (1H4 antibody; green), amyloid-β (red) and the amyloid dye Methoxy-X04 (cyan). Blood vessels show substantial medin staining (**a**,**b**) (quantification for 5 female and 5 male patients, with a total of *n* = 478 vessels analysed), while staining is absent on amyloid plaques (**c**). **d**, Confocal *z*-stack of an isolated human cerebral blood vessel and western blotting of the vascular marker α-SMA and MFG-E8. **e**, Quantification of MFG-E8 by ELISA (controls: 4 women and 5 men; Alzheimer’s disease with low CAA: 5 women and 5 men; Alzheimer’s disease with high CAA: 4 women and 5 men). AD, Alzheimer’s disease; ctrl, control. **f**, Quantification of total amyloid-β from the individuals in **e** by ELISA. **g**, Western blot analysis of different fractions using an anti-human medin antibody (6B3) (3 female and 3 male age-matched individuals per group). Note the very low protein levels in formic acid-extracted, RIPA-insoluble fractions, evident from the lack of β-actin signal. **h**, *MFGE8* expression in 566 individuals from the ROSMAP cohort^23^ (control: 123 women and 78 men; mild cognitive impairment (MCI): 94 women and 49 men; Alzheimer’s disease: 152 women and 68 men). Linear regression analysis was used to examine the effect of *MFGE8* expression levels, plaque load (CERAD score) and tau pathology (Braak score; numbers in brackets indicate a change between two particular scores) on cognitive ability (mmse30 score), indicating an independent contribution of *MFGE8* levels to cognitive dysfunction. The heat map shows WGCNA module 3 of the RNA-seq data set, which contains the *MFGE8* gene (green) and is associated with Alzheimer’s disease. Expression-weighted cell-type enrichment demonstrates that the increased *MFGE8* expression in patients with Alzheimer’s disease is attributable to smooth muscle cells (*P*_adj_ < 0.05; see Methods). Data are mean ± s.e.m. FPKM, fragments per kilobase million. **e**, Two-way ANOVA with Tukey’s post hoc test. **f**,**g**, Kruskal–Wallis test with Dunn’s post hoc test, **h**, with Benjamini–Hochberg correction. Scale bars: 20 µm. Uncropped western blots are shown in Supplementary Fig. 2. Source data

**Extended Data Fig. 7:**
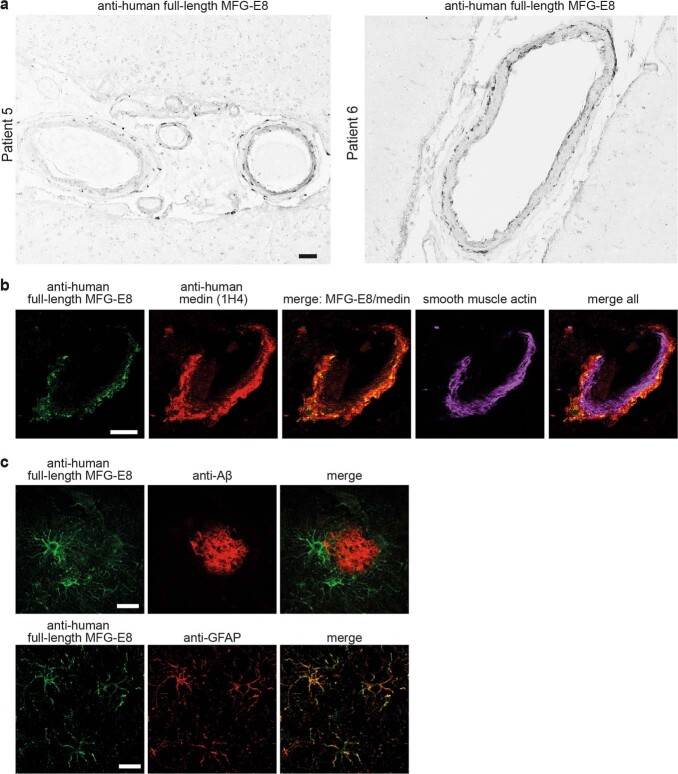
Immunostaining for full-length MFG-E8 shows cellular staining of vascular cells and astrocytes. **a**, Brain sections of two sporadic AD patients were stained with a monoclonal antibody raised against human full-length MFG-E8. **b**, Exemplary image of co-staining for human full-length MFG-E8 (green), and human medin (1H4, red) showing limited overlap between full-length MFG-E8 and medin in a cerebral blood vessel (stained with smooth muscle actin; tissue sections are from Patient 4). **c**, Staining for full-length MFG-E8 (green) in human AD brain sections also labels cells around amyloid plaques (anti-Aβ, red), which are identified as astrocytes by co-staining for glial fibrillary acidic protein (GFAP). Scale bars: 50 µm in *a*, 20 µm in *b*, 50 µm in *c*.

To assess the role of medin in human Alzheimer’s disease pathology, we examined postmortem frontal and occipital brain tissue from 16 patients with Alzheimer’s disease by immunostaining (patient information is presented in Supplementary Table 1). First, we stained the brain sections with an antibody against full-length human MFG-E8, which (in line with our mouse models) labelled astrocytes and also showed some localized vascular immunoreactivity (Extended Data Fig. 7). By contrast, an antibody raised against human medin^3^ (1H4) showed prevalent immunoreactivity on aggregate-like structures in the cerebral vasculature but did not detectably label amyloid plaques in any of the 16 patients (Figs. 2a–c), in line with the reported vascular localization of medin in humans^1,3,9^.

Next, we performed co-staining for human medin and amyloid-β alongside Methoxy-X04 staining, equivalent to our analysis in mouse models (Fig. 1d), using brain sections from 5 male and 5 female patients with Alzheimer’s disease and CAA (with a total of 478 individual vessels analysed). We found that around 49% of Methoxy-X04 staining co-localized with amyloid-β staining, whereas around 14% of Methoxy-X04 staining was co-labelled by the medin antibody. Similarly, around 39% of medin staining co-localized with Methoxy-X04 staining and around 33% of medin staining was co-labelled by the amyloid-β antibody. This suggested that medin also interacts with amyloid-β in both amyloid and pre-amyloid forms in the human brain vasculature.

To examine the relationship between medin and CAA in more detail, we next isolated cerebral blood vessels from occipital cortex samples from control patients (4 female and 5 male; 72.0 ± 4.4 years of age) and those from neuropathologically diagnosed patients with Alzheimer’s disease with no or mild CAA (5 female and 5 male; 73.0 ± 3.7 years of age) or from patients with Alzheimer’s disease with severe CAA (4 female and 5 male; 75.6 ± 2.9 years of age) (Supplementary Table 1). Reflecting our data from the mouse models, MFG-E8 protein levels were 30–40 times higher in cerebral blood vessels compared with total brain homogenates from the same individuals (Figs. 2d,e). Moreover, in blood vessels from patients with Alzheimer’s disease with high CAA versus those with no or only mild CAA (which we confirmed by analysis of amyloid-β levels; Fig. 2f), MFG-E8 levels were further increased by approximately 2.3-fold, again mirroring our findings in APP23 mice with low- versus high CAA pathology (Fig. 1i). Similarly, MFG-E8 levels also correlated positively with total amyloid-β levels and the ratio of Aβ40/Aβ42 in human blood vessels, with the strongest correlations found for the insoluble vascular fraction (Extended Data Fig. 6), confirming that increased MFG-E8 levels are associated with CAA in patients with Alzheimer’s disease.

Next, we used western blotting analysis to examine the amount of MFG-E8 and medin in total brain homogenate and soluble and insoluble vascular fractions from six age- and sex-matched individuals per group. Detection with an anti-human medin antibody confirmed that full-length MFG-E8 levels were higher in all fractions in patients with Alzheimer’s disease with high CAA compared with controls and patients with Alzheimer’s disease with low CAA. Moreover, only in patients with Alzheimer’s and high CAA, the soluble vascular fraction showed increased fragmentation of MFG-E8, whereas the insoluble vascular fraction (despite the more than 60-fold dilution resulting from formic acid extraction) showed a smear across the entire molecular weight range, indicating the presence of medin aggregates. Notably, the intensity of the medin signal reflected the amyloid-β content of the individual samples (Fig. 2g). These data provide direct evidence that increased MFG-E8 and medin levels are specifically associated with CAA in patients with Alzheimer’s disease.

## *MFGE8* is associated with cognitive decline

**Extended Data Fig. 8:**
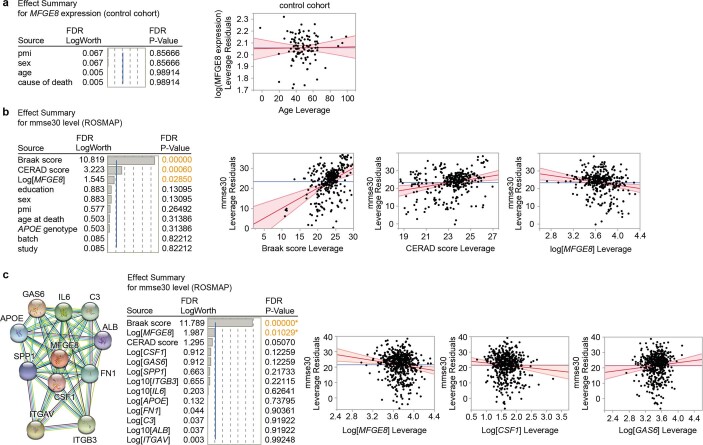
The impact of *MFGE8* gene expression on measures of cognitive decline in AD patients. Linear regressions (Standard Least Squares) were performed to analyse **a**, the impact of ageing on *MFGE8* gene expression in a control cohort of 116 patients who died without dementia, and **b**, the impact of *MFGE8* gene expression on cognitive performance (‘mmse30’) adjusted for Aβ plaque load (‘CERAD score’), tau pathology (Braak and Braak: ‘Braak score’), education level, post-mortem interval (‘pmi’), sex, age at death, *APOE* genotype, RNA-sequencing batch (‘batch’) as well as the study (ROS vs. MAP) for the data from 566 ROSMAP patients analysed in Fig. 2h. **c**, The impact of gene expression levels of *MFGE8* and 10 genes within its known molecular network (https://string-db.org/) on cognitive performance (‘mmse30’) was analysed in the same patients as in *b*, adjusted for Aβ plaque load (‘CERAD score’) and tau pathology (‘Braak score’). Selected regression plots are shown as so-called residual vs. leverage plots (see Methods for details): crossing of a least squares line (red) and 95% confidence bands (shaded red) through the mean of the Y leverage residuals (blue line) indicates statistical significance. FDR: False Discovery Rate (adjusted P-value using the Benjamini-Hochberg correction). FDR LogWorth: -log10(FDR P-Value) for each model effect.

**Extended Data Fig. 9:**
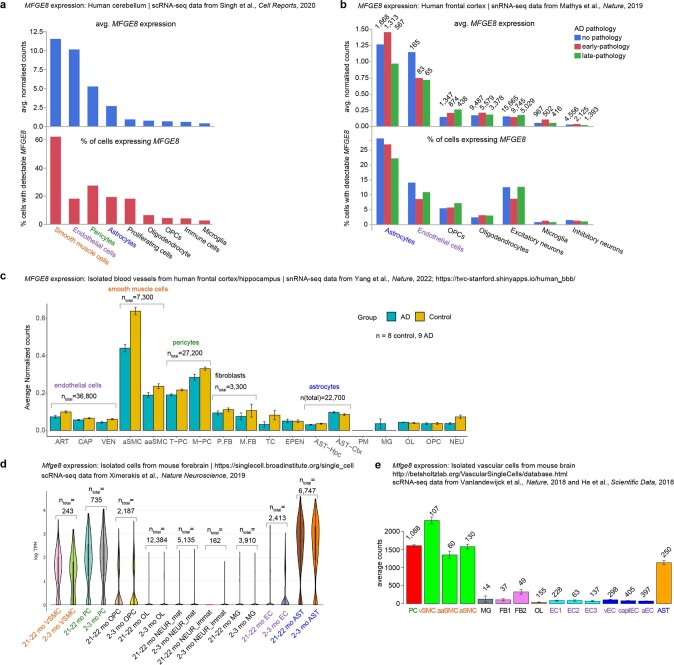
Cell type-specific *MFGE8*/*Mfge8* gene expression in the brain. Published single cell RNA-sequencing data sets of brain tissue were examined with regard to *MFGE8/Mfge8* expression levels (where available, the number of cells analysed is shown above the bars). **a**, In the human cerebellum, smooth muscle cells show the highest *MFGE8* gene expression (*top*) and the largest fraction of cells with detectable *MFGE8* levels (*bottom*)^69^. **b**, In the human frontal cortex from patients with no, early or late Alzheimer’s disease pathology, astrocytes show the highest *MFGE8* expression levels and the largest fraction of cells expressing *MFGE8*, but they show decreasing levels of *MFGE8* with AD pathology (note that only a small number of endothelial cells was captured in this study)^70^. **c**, In isolated blood vessels from the human brain, arterial smooth muscle cells (aSMC) show the highest levels of *MFGE8*^71^ (corroborating our analysis of the ROSMAP data in Fig. 2). While there appears to be a down-regulation of *MFGE8* expression in aSMCs in AD patients, it is unknown if these patients had CAA pathology. **d**, In the mouse forebrain, vascular smooth muscle cells (VSMC), pericytes and (in contrast to human brain) also astrocytes show the highest *Mfge8* expression levels, which appear to be unaffected by ageing itself^72^ (similar to our findings in human tissue; cf. Extended Data Fig. 8a). **e**, In isolated blood vessels from the mouse brain, smooth muscle cells and pericytes and again also astrocytes show the highest *Mfge8* gene expression levels^73,74^. ART: Arterial; CAP: Capillary; VEN: Venous; vSMC/aSMC: Vascular Smooth Muscle Cell; aaSMC: Arteriolar Smooth Muscle Cell; PC: Pericyte; T-PC: Solute Transport-Pericyte; M-PC: ECM-regulating Pericyte; FB: Fibroblast; P.FB: Perivascular Fibroblast; M.FB: Meningeal Fibroblast; TC: T cell; EC: Endothelial cell; EPEN: Ependymal; AST: astrocyte; AST-Hpc: Astrocyte-Hippocampus; AST-Ctx: Astrocyte-Cortex; PM: Perivascular Macrophage; MG: Microglia; OL: Oligodendrocyte; OPC: Oligodendrocyte Precursor Cell; NEU: Neuron; mat: mature; immat: immature.

Having established that MFG-E8 is highly enriched in the brain vasculature and that its levels are strongly associated with CAA in patients with Alzheimer’s disease, we then assessed the relevance of our findings for dementia. To this end, we examined *MFGE8* gene expression in dorsolateral prefrontal cortex samples from 566 patients in the ROSMAP cohort^23^ (focussing on those with Alzheimer’s disease as the only known cause of cognitive impairment—that is, final clinical consensus diagnosis of cognitive status (cogdx) 1 versus 2 versus 4 (ref. ^24^)). Notably, *MFGE8* expression was significantly increased in patients with Alzheimer’s disease (85.9 ± 3.7 years of age; 69% female) compared with controls without dementia (82.9 ± 5.0 years of age; 61% female) (Fig. 2h and Supplementary Table 2). By contrast, *MFGE8* expression did not vary in an age-dependent manner in frontal lobe samples from a control cohort of 116 individuals between 15 and 95 years of age^25^ (Extended Data Fig. 8a and Supplementary Table 2), demonstrating that the increase in *MFGE8* expression occurred as a result of Alzheimer’s disease-associated pathology rather than age. Of note, we found a significant association of higher *MFGE8* expression levels with increased measures of cognitive decline in the ROSMAP data (as measured by the Mini Mental State Examination (mmse30) test battery) that was independent of amyloid-β plaque load (CERAD score) and tau pathology (Braak score) (Fig. 2h). This effect also remained significant when the model was adjusted for age, sex, education, postmortem interval, *APOE* genotype, RNA-sequencing batch and the ROS versus MAP studies (Extended Data Fig. 8). Moreover, ten other genes from the reported molecular network of MFG-E8 showed no significant association with cognitive decline (Extended Data Fig. 8). To examine the source of increased *MFGE8* expression in patients with Alzheimer’s disease, we further interrogated the RNA-sequencing data by constructing gene regulatory networks using SCENIC^26^ to identify genes that are co-regulated with *MFGE8*. This revealed only one group (or module) of genes associated with Alzheimer’s disease pathology (that is, with at least ten genes being differentially expressed in Alzheimer’s disease patients versus controls) (Fig. 2h). Using expression weighted cell type enrichment analysis^27^, we then tested whether genes within this module could be attributed to a particular cell type; this identified smooth muscle cells as the only significant hit (adjusted *P*-value (*P*_adj_) < 0.05), with endothelial cells being the second most likely source (*P*_adj_ < 0.1). Thus, these data indicate that increased *MFGE8* gene expression is most probably driven by vascular alterations in patients with Alzheimer’s disease. These results are in line with our analysis of MFG-E8 protein levels in human brain samples and are further supported by recent single-cell analyses demonstrating that the main cells expressing *MFGE8* in the human brain are smooth muscle cells and pericytes, both in terms of expression level and the fraction of cells expressing *MFGE8* (Extended Data Fig. 9). Of note, equivalent studies in mice show high expression levels in smooth muscle cells and pericytes, but also in astrocytes (Extended Data Fig. 9), providing an explanation for why medin may affect parenchymal amyloid-β deposits in mouse models (owing to high astrocytic expression) but cannot be detected in human plaques (owing to lower astrocytic expression).

## Medin directly promotes amyloid-β aggregation

**Fig. 3:**
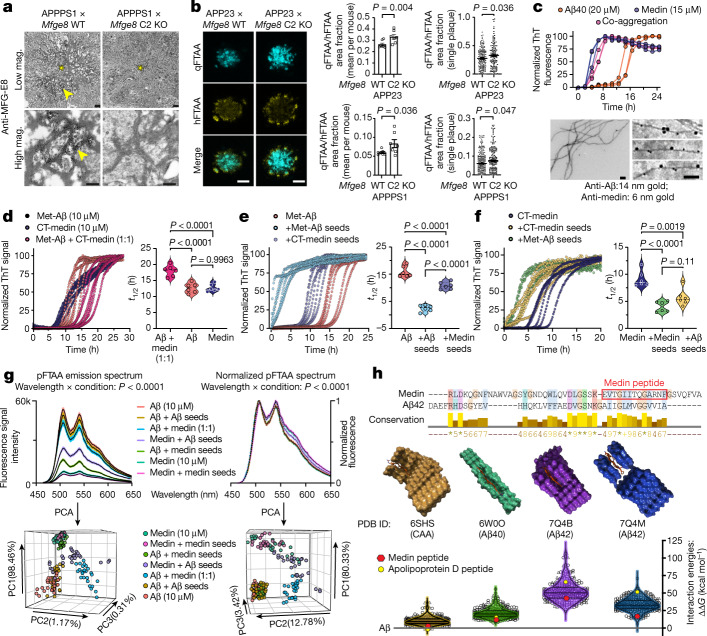
Medin interacts directly with amyloid-β, promoting its aggregation. **a**, Immuno-electron microscopy of MFG-E8 (or fragment) staining in cortical brain sections from 4-month-old APPPS1 × *Mfge8* wild-type or APPPS1 × *Mfge8* C2 KO mice. Asterisks indicate the plaque core and arrowheads show MFG-E8 staining on amyloid fibrils (APPPS1 ×* Mfge8* wild-type: 1 female and 1 male mouse; APPPS1 ×* Mfge8* C2 KO: 1 female and 1 male mouse). **b**, Amyloid staining with LCOs for compact (qFTAA) and diffuse (hFTAA) amyloid, and quantification of their area ratio in 12-month-old APP23 and 4-month-old APPPS1 mice (APPPS1 ×* Mfge8* wildtype: 3 female and 4 male mice; APPPS1 × *Mfge8* C2 KO: 3 female and 4 male mice; APP23: 167 * Mgfe8* wild-type and 131 C2 KO plaques; APPPS1: 244 wild-type and 206 C2 KO plaques). **c**, Top, in vitro ThT-based co-aggregation assay using recombinant Aβ40 and medin (*n* = 3 independent experiments). Bottom, immuno-electron microscopy analysis of co-aggregated peptides. **d**–**g**, In vitro ThT assay using methionine-Aβ42 (Met-Aβ, 10 µM) and a C-terminal medin fragment (CT-medin, 10 µM) for co-aggregation of monomeric peptides (**d**) and induction of Met-Aβ (**e**) or CT-medin (**f**) aggregation with 0.5 µM pre-formed seeds. *t*_1/2_, aggregation half-time. **g**, Structural properties of aggregates were assessed on the basis of raw or normalized emission spectra of the LCO pFTAA (black line shows mean and coloured area indicates s.e.m.) and the PCA of these spectra (*n* = 3 independent experiments, with 2 replicates each). **h**, Structural fitting of a medin peptide with high homology to amyloid-β (red box) interacting with reported amyloid-β assemblies—with indicated PDB IDs—derived from Alzheimer’s disease brains. Top, alignment of medin and Aβ42 amino acid sequences, showing conservation levels. Asterisks indicate identical amino acids. Middle, graphical model of the interaction of the medin peptide (red) with amyloid-β structures. Bottom, fibril stability values for each amyloid-β aggregate structure interacting with random peptides (triangles; *n* = 100) and peptide sequences from blood proteins (circles; *n* = 250) compared with amyloid-β itself (that is, free energy (ΔΔ*G*) for amyloid-β is zero). Violin plots show median and quartiles. Bar charts display mean ± s.e.m. **b**, One-tailed Mann–Whitney *U*-test. **d**–**g**, One-way ANOVA with Tukey’s post hoc test. Mag., magnification; scale bars: 1 μm (**a**, top), 250 nm (**a**, bottom)*,* 40 µm (**b**), 200 nm (**c**). Source data

The co-localization of medin and amyloid-β in blood vessels of both mouse and human brain, and the persistent reduction of CAA pathology in medin-deficient mice suggested that the two amyloids might be interacting directly. To examine this further, we first analysed the subcellular localization of medin and MFG-E8 around amyloid-β plaques using immuno-electron microscopy on brain tissue from APPPS1 × *Mfge8* wild-type or APPPS1 × *Mfge8* C2 KO mice. In APPPS1 × *Mfge8* wild-type mice, we found conspicuous immunolabelling of amyloid fibrils, which was absent in APPPS1 × *Mfge8* C2 KO mice (Fig. 3a), in line with our immunohistochemical analysis (Fig. 1). Moreover, amyloid plaques showed a less pronounced fibril structure in APPPS1 × *Mfge8* wild-type compared mice with APPPS1 x *Mfge8* C2 KO mice (Fig. 3a), indicating that medin—which frequently forms amorphous rather than fibrillar aggregates in vivo^3^—may induce a less ordered structure of amyloid deposits. To quantify this change, we used two conformation-sensitive amyloid dyes—luminescent conjugated oligothiophenes^28^ (LCOs)—to stain tissue sections. For this analysis, we focussed specifically on age groups with similar plaque burden in *Mfge8* wild-type and C2 KO mice (namely 4-month-old APPPS1 and 12-month-old APP23 mice; Fig. 1e,f) to determine whether medin would affect amyloid-β aggregation independently of a change in plaque load, and to avoid confounding effects resulting from plaque size, which affects LCO affinity^29^ (average plaque size analysed in APPPS1 × *Mfge8* wild-type and APPPS1 × C2 KO mice: 695 ± 209 and 783 ± 170 arbitrary units (AU), respectively; APP23 × *Mfge8* wild-type and APP23 × C2 KO mice: 1,549 ± 72 and 1,554 ± 65 AU, respectively). Indeed, a significant change towards more compact amyloid was evident in medin-deficient mice, both in the average value per mouse as well as for individual plaques (Fig. 3b), consistent with our immuno-electron microscopy analysis.

Amyloid compaction can also be altered by microglia, which may in turn affect neuritic dystrophy^30^, and full-length MFG-E8 is best known for its function as an opsonin, mediating phagocytic uptake by macrophages^31^. We therefore analysed microglial plaque association and plaque-associated neuritic damage, but did not detect *Mfge8* genotype effects in either mouse model (Extended Data Fig. 4). This indicated that medin influences amyloid-β aggregation independently of microglial function. We next tested whether recombinant amyloid-β and medin would co-aggregate in vitro, using a Thioflavin T (ThT) affinity assay. Medin (15 µM) aggregated significantly faster than Aβ40 (20 µM), whereas a mixture of medin and Aβ40 aggregated with the same rate as medin alone and did not exhibit a biphasic ThT fluorescence profile (Fig. 3c), as might be expected if the peptides were aggregating independently. Moreover, immuno-electron microscopy of the aggregated material showed labelling for both medin and amyloid-β on individual amyloid fibrils (Fig. 3c), indicating co-aggregation.

As the full-length medin peptide aggregated rapidly both with and without Aβ40 in vitro, we used two modified peptides with slower aggregation kinetics (Fig. 3d–g) to further examine their interaction in vitro, namely methionine-Aβ42 (Met-Aβ) and a C-terminal fragment of medin (CT-medin, EVTGIITQGARNFGSVQFVASYK) that we most consistently identified in tissue extracts by mass spectrometry analyses^3^. We incubated the peptides in a 1:1 mixture or used pre-formed aggregates (‘seeds’) of either peptide to assess whether they could accelerate each others’ aggregation. Similar to the full-length peptides, Met-Aβ and CT-medin readily co-aggregated, albeit with slightly slower kinetics than the individual peptides alone (Fig. 3d). Moreover, seeds of either peptide (0.5 µM) accelerated aggregation of the homologous peptide as well as the heterologous peptide, although Met-Aβ seeds induced more rapid seeding of Met-Aβ aggregation than did CT-medin seeds (Figs. 3e,f). Similar to our in vivo analysis of plaque morphotypes (Fig. 3b), we then used an LCO to examine the amyloid structure of the resulting aggregates. We observed that our experimental conditions affected both the normalized LCO emission spectrum and its amplitude. Principal component analysis (PCA) of either the raw data (preserving amplitude as a factor) or of the normalized spectra (focussing on changes in relative emission values) clearly separated the equimolar co-aggregates of Met-Aβ and CT-medin from other conditions (Fig. 3g). Moreover, the amplitudes of the raw emission spectra were noticeably lower for CT-medin than for Met-Aβ aggregates, with values being virtually identical when either peptide aggregated autonomously or in response to homologous seeding. By contrast, seeding with Met-Aβ clearly shifted CT-medin aggregates towards a higher LCO emission amplitude, whereas seeding with CT-medin reduced the LCO amplitude of amyloid-β aggregates, indicating that the seed affected the amyloid structure of the heterologous peptide. This finding was also reflected in the PCA, in which Met-Aβ seeded with CT-medin clustered tightly with both autonomously aggregating and homologously seeded CT-medin (Fig. 3g, left). When analysing the normalized spectra, however, seeding Met-Aβ or CT-medin with either homologous or heterologous seeds resulted in aggregates that were indistinguishable from their unseeded peptides alone (Fig. 3g, right). Thus, heterologous seeding of medin and amyloid-β differentially affects LCO emission amplitudes (largely reflecting the seed) and normalized spectra (largely reflecting the major aggregating species), whereas equimolar co-aggregation yields distinct spectra for both analyses.

Next, we used structural modelling as an independent approach to interrogate the interaction of amyloid-β and medin. Using the recently determined structures of amyloid-β fibrils purified from human Alzheimer’s disease brains—assemblies of Aβ42^32^ (Protein Data Bank identifiers (PDB IDs): 7Q4B and 7Q4M), Aβ40^33^ (PDB ID: 6W0O) and CAA^34^ (PDB ID: 6SHS)—we modelled their interaction with a shorter peptide segment of CT-medin (EVTGIITQGARNF) with high homology to amyloid-β (Fig. 3h). We compared the resulting interaction energies with a set of random peptides of equal length extracted from blood protein sequences (*n* = 250) or completely random peptide sequences (*n* = 100). First, we observed that two peptides from blood proteins achieved lower interaction energies than amyloid-β itself when interacting with the CAA aggregate structure (6SHS). Notably, the top hit was a peptide derived from apolipoprotein D, a reported biomarker for CAA and a modulator of amyloid-β aggregation^35,36^, and the second hit was complement factor D^37^, another potential biomarker for Alzheimer’s disease—indicating that our modelling approach can identify physiologically relevant peptides. Indeed, the energies of the CT-medin fragment ranked in the top 5% of the 350 peptides for three out of the four amyloid-β fibril polymorphs (ranking 8th for 6W0O, 10th for 6SHS, 16th for 7Q4M and 74th for 7Q4B). Notably, the interaction energies of the medin peptide with the CAA and Aβ40 assemblies approached the values for amyloid-β itself (Fig. 3h), indicating high structural compatibility and supporting a favourable interaction of medin, particularly with vascular amyloid-β aggregates.

Thus, our three independent data sets—using (1) ex vivo analyses of amyloid plaque structure, (2) co-aggregation and seeding of recombinant amyloid-β and medin peptides and (3) structural modelling—indicate that amyloid-β and medin are able to interact directly, promoting amyloid aggregation.

## Medin accelerates β-amyloidosis in vivo

**Fig. 4:**
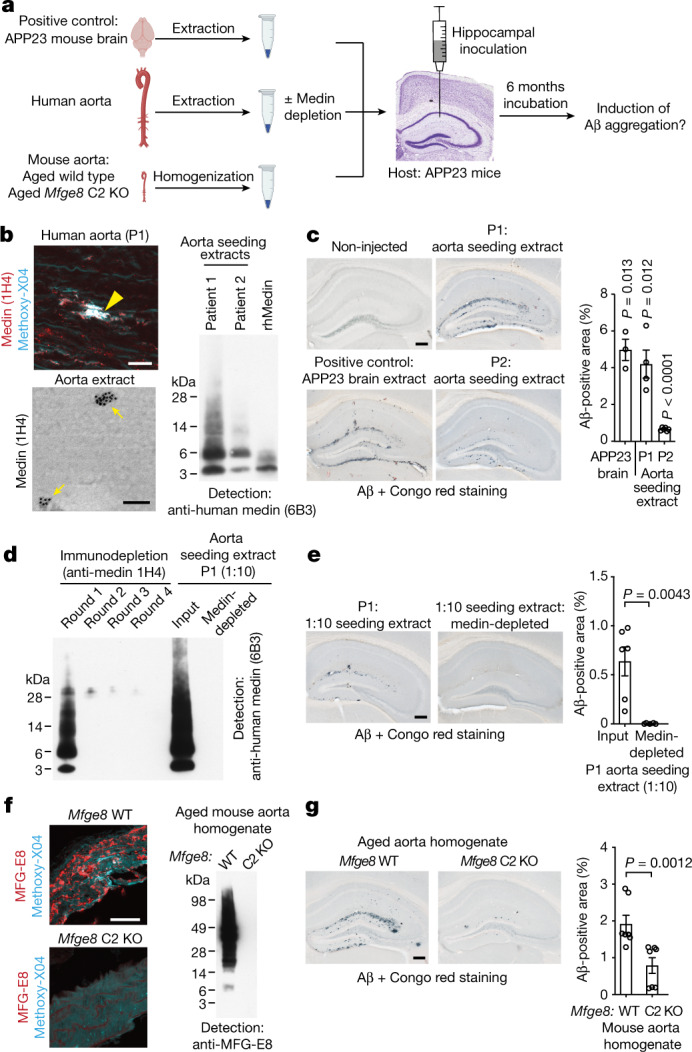
Exogenous medin aggregates induce premature amyloid-β aggregation in vivo. **a**, Experimental design to assess premature induction (seeding) of amyloid-β aggregation by medin aggregates in vivo. **b**, Top left, human aorta section from patient 1 (P1) stained for medin and amyloid (Methoxy-X04; note that collagen fibres are autofluorescent), showing a prominent medin deposit (yellow arrowhead). Bottom left, immuno-electron microscopy of human aorta extract. Right, western blotting of aorta extracts and recombinant human medin (rhMedin) using an anti-human medin antibody (6B3). **c**, Analysis of cerebral β-amyloidosis six months after intrahippocampal injection. Representative images of amyloid-β deposition induced by APP23 mouse brain extract (positive control) and aorta extracts from patients 1 and 2, compared with an age-matched un-injected APP23 mouse, which shows no endogenous deposits. Right, stereological quantification of amyloid-β deposition (APP23 mouse brain extract: *n* = 3; patient 1 extract: *n* = 4; patient 2 extract: *n* = 6 mice). **d**, Patient 1 aorta extract was diluted 1:10 (for technical reasons) and medin was immunodepleted using anti-human medin 1H4 antibody in four rounds of incubation. Depletion was examined by western blotting with 6B3 antibody. **e**, Quantification of amyloid-β seeding (extract: *n* = 6; depleted extract: *n* = 5 mice). **f**, Left, aged mouse aorta stained for MFG-E8 (medin) and amyloid (Methoxy-X04, mostly showing autofluorescence). Right, western blotting for MFG-E8 in aged *Mfge8* wild-type and *Mfge8* C2 KO aortas. **g**, Quantification of amyloid-β seeding with mouse aorta extracts (*Mfge8* wild-type aorta homogenate: *n* = 7 mice; *Mfge8* C2 KO aorta homogenate: *n* = 7 mice). Data are mean ± s.e.m. **c**, One-sample *t*-test against 0. **e**,**g**, Two-tailed Mann–Whitney *U*-test. Scale bars, 25 µm (**b**, top, **h**), 100 nm (**b**, bottom); 250 µm in (**c**,**f**,**i**). Uncropped western blots are shown in Supplementary Fig. 3. Source data

**Extended Data Fig. 10:**
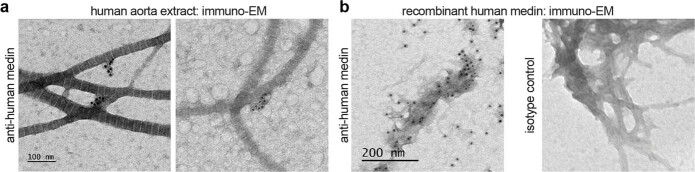
Medin aggregates extracted from human aorta. **a**, Immuno-electron microscopic analysis of amyloid extract from human aorta occasionally shows medin aggregates still associated with collagen and/or elastic fibres. **b**, Immuno-gold labelling of recombinant medin is only observed with the medin-specific 1H4 antibody (*left*) but not with an isotype control antibody (*right*).

Finally, we examined whether medin aggregates would also be able to seed amyloid-β pathology in the much more complex in vivo setting, similar to the ability of aggregated amyloid-β to induce premature cerebral β-amyloidosis^38^ (Fig. 4a). Here, we assessed the seeding efficacy of medin-containing aorta-derived material compared with homologous seeding with amyloid-β, using brain extract from an end-stage APP23 transgenic mouse (note that we have shown repeatedly that vehicle injections do not induce detectable amyloid-β seeding^38,39^). As expected, the intrahippocampal injection of APP23 mouse brain extract resulted in overt amyloid-β deposition six months later, whereas no endogenous amyloid-β plaques formed in the hippocampus of host mice of the same age (Fig. 4c). To model the in vivo situation as closely as possible, we then used tissue-derived medin aggregates to examine heterologous amyloid-β seeding. To this end, we extracted aggregated medin from two human aorta samples from a 69-year-old female and a 67-year-old male patient with substantial aortic medin deposition (Fig. 4b and ref. ^3^). Analysis of these extracts by immuno-electron microscopy revealed numerous particles 50–100 nm in diameter that were strongly labelled by an anti-human medin antibody (Fig. 4b), and were occasionally still attached to collagen or elastic fibres, reflecting their tissue localization (Extended Data Fig. 10). Hippocampal injection of these aortic extracts induced significant, premature amyloid-β aggregation 6 months later (Fig. 4c). Indeed, for one of the aorta samples, the extent of induced hippocampal amyloid-β deposition was similar to the effect of the aged APP23 brain extract—the lower seeding efficacy of the second sample reflected its lower medin content (Fig. 4c). To confirm that medin species were responsible for amyloid-β seeding, we depleted medin from the highly seeding-active aorta extract using the 1H4 antibody; this resulted in a complete absence of medin in the extract (Fig. 4d) as confirmed by western blotting using 6B3, a second anti-medin antibody) and fully abolished amyloid-β seeding (Fig. 4e). Previous reports found small amounts of amyloid-β in the human aorta^40,41^; however, using highly sensitive ELISA measurements, we were unable to detect amyloid-β in formic acid extracts of the human aorta samples used for injections (data not shown). Therefore, these results indicate that human medin aggregates are capable of inducing human amyloid-β aggregation in vivo, corroborating our in vitro and in silico analyses.

Since it is possible that amyloid extraction and immuno-depletion approaches are not entirely specific for medin, we also injected aorta samples from (APP non-transgenic) aged *Mfge8* wild-type or aged *Mfge8* C2 KO mice using total aorta homogenates to avoid any potential confounds introduced by extraction procedures (Fig. 4f). Corroborating our human medin depletion experiments, aorta homogenate from aged *Mfge8* wild-type mice was significantly more potent in seeding amyloid-β deposition than aorta homogenate from aged *Mfge8* C2 KO mice, although some nonspecific seeding was observed using these complex samples (Figs. 4f,g). Thus, tissue-derived medin aggregates can accelerate amyloid-β pathology via a heterologous seeding mechanism in vivo, confirming the pronounced interaction of these amyloids.

## Discussion

Despite its exceedingly high prevalence in the ageing population^2^, it is unknown how medin amyloid is generated, and it remained poorly understood whether it contributes to disease. Our recent work has demonstrated that during ageing, medin deposition leads to vascular stiffening in the brain of wild-type mice^3^. Two independent studies on postmortem human tissue further indicated that medin levels are increased in brain arterioles of patients with vascular dementia and Alzheimer’s disease^8,9^, but it was unclear whether medin contributes to brain dysfunction and how it relates to other pathological hallmarks of Alzheimer’s disease. Here we provide evidence for a direct interaction between amyloid-β and medin, demonstrating that medin readily co-aggregates with amyloid-β in vitro, cross-seeds amyloid-β aggregation both in vitro and in vivo (Figs. 3 and 4) and that a lack of medin in mouse models strongly reduces CAA burden and, as a result, damage to the brain vasculature (Fig. 1). Remarkably, endogenous levels of medin were able to influence amyloid-β aggregation in vivo, despite the substantial overexpression of APP in these mouse models^13,14^. Thus, our findings indicate a highly favourable interaction between medin and amyloid-β that promotes amyloid-β aggregation.

This amyloid–amyloid interaction may be explained by the fact that human medin contains an amino acid sequence that is homologous to amyloid-β located in its aggregation-prone C-terminal region (refs. ^42,43^ and Fig. 3h). Indeed, structural modelling revealed the high compatibility of the amyloid-β-homologous peptide of medin with amyloid-β fibril assemblies derived from human Alzheimer’s disease brains. Notably, there was minimal structural incompatibility (compared to amyloid-β itself) with an assembly extracted from vascular amyloid-β deposits, corroborating our histological data demonstrating co-localization of amyloid-β and medin in cerebral blood vessels both in mouse modelsand in human Alzheimer’s disease tissue (Figs. 1 and 2). Although we also found strong co-localization of MFG-E8 staining with amyloid-β plaques in APP transgenic mice, we were unable to detect medin staining on parenchymal amyloid-β deposits in the brains of patients with Alzheimer’s disease, possibly owing to the differences in astrocytic *MFGE8* expression levels between mouse (high expression) and human (low expression) compared with the high expression by vascular cells in both species (Extended Data Fig. 9).

Thus, it currently appears most likely that in the human brain, the contribution of medin to Alzheimer’s disease pathogenesis is driven by its effects on the vasculature. Accordingly, we report here that in a dataset including 566 patients, higher *MFGE8* expression levels are associated with increased measures of cognitive decline, even when adjusted for plaque and tau pathology, and that differentially higher expression of *MFGE8* in the same dataset is most probably driven by vascular cells in patients with Alzheimer’s disease (Fig. 2h). Supporting this hypothesis, there is no genetic evidence that would directly link MFG-E8 or medin to Alzheimer’s disease; however, expression quantitative trait loci have been identified that enhance or reduce *MFGE8* expression levels and thereby increase and decrease the risk of vascular disease, respectively^44–47^. As we here report strong correlations of cerebrovascular MFG-E8 levels with CAA burden (both in mouse and human brain tissue; Extended Data Fig. 6), it is conceivable that genetically driven increases of MFG-E8 levels eventually cause medin deposition that in turn promotes CAA and vascular damage. Accordingly, we found not only higher levels of MFG-E8 but also increased MFG-E8 fragmentation and medin-containing species specifically in patients with Alzheimer’s disease with high CAA burden (Fig. 2g). As it is becoming increasingly clear that Alzheimer’s disease pathogenesis has an important vascular component^21,48–50^, medin could significantly contribute to cognitive decline in Alzheimer’s disease by altering vascular function and amyloid-β deposition. In view of our data demonstrating a role for medin in driving age-associated vascular dysfunction^3^ as well as CAA (in this Article), we therefore propose that targeting medin might provide a novel therapeutic approach to preserving brain function during ageing and Alzheimer’s disease by improving vascular health.

## Methods

### Human tissue

Ascending aortic tissue samples were obtained from patients undergoing elective aneurysmal repair at Liverpool Heart and Chest Hospital (Supplementary Table 1). This study was ethically approved by Liverpool Bio-Innovation Hub (project approval reference 15-06 and 18-07), and informed consent was obtained for all participants. The LBIH Biobank confers ethical approval for the use of samples through their ethical approval as a Research Tissue Bank (REC reference 14/NW/1212, NRES Committee North West–Haydock). After collection, samples were rapidly frozen in dry ice and isopentane slurry, and immediately stored at –80 °C prior to use.

Human brain tissue (Supplementary Table 1) was obtained from the Queen Square Brain Bank for Neurological Disorders (UCL Institute of Neurology, London, UK; approval protocol no. EXTMTA5/16) and the Emory University Alzheimer’s Disease Research Center (IRB 00045782) with informed consent from families. FFPE brain sections from the occipital, frontal or temporal cortex were used for analysis (Supplementary Table 1).

This study was also approved by the ethical committee of the Medical Faculty, University of Tübingen, Germany (protocols 354/2016BO2, 832/2021BO2 and 369/2021BO2).

### RNA-sequencing data of human brain samples

RNA-sequencing data from human brain were from the ROSMAP study^23^. Study data were provided by the Rush Alzheimer’s Disease Center, Rush University Medical Center, Chicago. Additional phenotypic data can be requested at www.radc.rush.edu.

### Mice

Male and female C57BL/6J and *Mfge8* C2 knockout (C57BL/6J-Mfge8 Gt^(KST227)Byg^) mice^15^ (provided by C. Théry) were bred in-house. *Mfge8* C2 KO mice were crossed with hemizygous APP transgenic mice. The APPtransgenic mouse lines used were APPPS1 (C57BL/6J-Tg(Thy1-APP_K670N;M671L_ and Thy1-PS1_L166P_; generated on a C57BL6/J background)^13^, and APP23 (C57BL/6J-Tg(Thy1-APP_K670N;M671L_)^14^ and C57BL/6J-Tg(Thy1-APPDutch)^17^; backcrossed with C57BL/6J for more than 20 generations. For some experiments (such as comparisons between APP23 age groups and in vivo inoculations), mice from a separate line of APP23 mice were also used (C57BL/6JNpa-Tg(Thy1App)23/1Sdz); no differences in any measures of pathology were apparent in our experiments between these two lines (which are derived from the same founder line), as reported also previously^51^. Where possible, littermate controls were used. All mice were maintained under specific pathogen-free conditions. Experiments were performed in accordance with German veterinary office regulations (Baden-Württemberg) and were approved by the local authorities for animal experimentation (Regierungspräsidium) of Tübingen, Germany (Approval numbers: N03/14, N02/15, N03/15, N07/16, N3/19, §4MIT v. 05.03.2018, §4MIT v. 18.08.2016; N06/21M).

### Tissue preparation

For mouse tissue preparations, mice were deeply anaesthetized and transcardially perfused with phosphate-buffered saline (PBS). Brains were fixed for 24 h in 4% paraformaldehyde (PFA). Optionally, one hemisphere was fresh-frozen on dry ice for biochemical analysis. The PFA-fixed hemisphere was then transferred to 30% sucrose for 48 h, and subsequently frozen in 2-methylbutane before long-term storage at −80 °C. Coronal sections of 25 µm were cut with a freezing sliding microtome (Leica). After removal of the perivascular adipose tissue, the aorta was freshly frozen on dry ice.

### Protein extraction

For downstream biochemical analysis, tissue samples were homogenized using a Precellys instrument (Bertin Instruments; two times for 10 s at 5,500 rpm for brain and six times for 30 s at 6,000 rpm for aorta) for 10% or 20% (w/v) brain homogenates in Tris-HCl buffer (50 mM Tris pH 8, 150 mM NaCl, 5 mM EDTA, phosphatase and protease inhibitors (Pierce)) or 10% (w/v) aortic homogenates in PBS. Total protein concentration of homogenates was quantified using the BCA assay (Pierce, Thermo Fisher) according to standard protocols.

In wild-type mice, soluble amyloid-β was extracted from 20% brain homogenates by adding an equal volume of 0.4% diethylamine (DEA) (in 100 mM NaCl) followed by rigorous vortexing. After ultracentrifugation for 1 h at 135,000*g* (fixed-angle TLA-55 rotor, Beckman Coulter) at 4 °C, the supernatant was neutralized with 0.5 M Tris-HCl (pH 6.8, 1:10 ratio) and flash-frozen on dry ice.

For extraction of insoluble protein aggregates in whole brain, RIPA-soluble and insoluble vessel fraction or isolated microglia (50,000 cells) from APP transgenic mice and human tissue, samples were treated with formic acid (Sigma-Aldrich, final concentration 70% v/v), sonicated for 30 s on ice and centrifuged at 25,000*g* for 1 h at 4 °C. Supernatants were then mixed with neutralization buffer (1 M Tris base, 0.5 M Na_2_HPO_4_, 0.05% NaN_3_ w/v; 1:20 ratio) for further downstream biochemical analysis.

### Microglial isolation and in vivo phagocytosis assay

One day before microglial isolation, mice were intraperitoneally injected with 17.5 μl per g body weight of the amyloid dye Methoxy-X04 (4% vol of 10 mg ml^−1^ Methoxy-X04 in DMSO, 7.7% vol CremophoreEL in PBS). Microglia were isolated as previously described^52^. In brief, the neocortex was dissected and minced in ice-cold Hanks buffered salt solution (HBSS) (15 mM HEPES, 0.54% d-glucose, 0.1% DNase w/v). The minced tissue was sequentially homogenized in glass Dounce and Potter homogenizers (Wheaton). Tissue suspension was filtered through a 70-μm cell strainer (BD Biosciences) and centrifuged at 300*g* for 15 min at 4 °C in a swinging-bucket rotor. The pellet was resuspended in 70% Percoll solution (Healthcare) and centrifuged for 30 min at 800*g* at 4 °C through a 70%, 37% and 30% isotonic Percoll gradient.Cells were recovered from the 70%–37% interphase and washed with fluorescence-activated cell sorting (FACS) buffer (1× HBSS, 2% FCS, 10 mM EDTA) by centrifugation at 300*g* for 15 min at 4 °C. For blocking of nonspecific Fc receptor-mediated antibody binding, the cell pellet was resuspended in FACS buffer, and Fc-block (BD, 1:400) was added for 10 min. Cells were stained with anti-mouse CD45 A700 (BioLegend, 1:200) or anti-mouse CD45 FITC (Affymetrix Bioscience, 1:100) and anti-CD11b APC (BioLegend, 1:200) for 15 min at 4 °C. After washing, the pellet was resuspended in FACS buffer containing 25 mM HEPES. CD11b^high^CD45^intermediate^ microglial cells were sorted with a Sony SH800 flow cytometer (Sony software, v 2.1.5) in FACS buffer containing 25 mM HEPES. Isolated cells were pelleted (800*g* for 7 min) and stored at −80 °C.

For quantification of amyloid-β phagocytosis in vivo, microglia were isolated and the proportion of Methoxy-positive microglia was analysed by flow cytometry with MACSQuant Analyzer (Miltenyi Biotec; MACSQuantify, v. 2.11). Background signals were excluded by gating based on APP non-transgenic mice for each separate experiment. APPPS1 × *Mfge8* C2 KO signals were normalized to the APPPS1 × *Mfge8* C2 wild-type mean of each experiment to reduce batch effects. The Methoxy-positive fraction of APPPS1 × *Mfge8* C2 KO was normalized to the mean of the entire experimental wild-type group. Additionally, phagocytosed amyloid-β was measured in isolated microglia by SIMOA Human Aβ42 2.0 Kit (Quanterix) after formic acid extraction (see sections ‘Protein extraction’ and ‘ELISA’), following the manufacturer’s protocol.

### Cerebral blood vessel isolation

Cerebral blood vessels were isolated from frozen mouse and human brain following published protocols^53–56^, with small modifications. For mouse brain, 600 or 200 μl of 10% brain homogenate (see ‘Protein extraction’) were used to isolate vessels from brain. For isolation of human cerebral vessels, 200 mg tissue (grey and white matter without prior removal of meninges) was cut from frozen samples of the occipital cortex and freshly homogenized (10% w/v) in HBSS (NaCl, KCl, KH_2_PO_4_, glucose) with 10 mM HEPES. Homogenates were centrifuged in a fixed-angle bucket rotor at 2,000*g* for 10 min at 4 °C. The supernatant was removed and mixed with an equal volume of 2× RIPA buffer (1× RIPA: 10 mM Tris, pH 8.0; 1 mM EDTA; 1% Triton X-100; 0.1% sodium deoxycholate; 0.1% SDS; 140 mM NaCl) freshly supplemented with protease and phosphatase inhibitors (Pierce). The pellet containing the vessel fraction was resuspended in 18% (w/v) dextran solution (HBSS, 10 mM HEPES; Dextran 70,000 MW, Roth), mixed and centrifuged at 4,400*g* for 15 min at 4 °C. The resulting supernatant, including the myelin layer, was removed, and the pellet was resuspended in HBSS buffer (supplemented with 1% w/v bovine serum albumin, BSA). The vessel suspension was filtered through a 20-μm (for mouse vessels) or 40-µm (for human vessels) PET mesh (pluriStrainer), washed twice and recovered from the inverted strainer with 20 ml HBSS buffer with 1% BSA. The purified vessels were centrifuged for 20 min at 4,400*g* (mouse) or 2,000*g* (human) at 4 °C. The supernatant was aspirated, and the isolated vessels were resuspended in 1 ml BSA-HBSS and transferred to a 1.5 ml tube. To verify successful vessel isolation with our protocol, one drop of the isolated vessels suspension was dried on poly-d-lysine-precoated coverslips, fixed with 4% PFA for 15 min at room temperature and stained for vascular and amyloid markers.

To remove BSA before analysis, vessels were centrifuged again for 15 min at 10,000*g* and 4 °C and resuspended in 1 ml of HEPES-HBSS buffer, followed by centrifugation for 10 min at 10,000*g* and 4 °C. The resulting pellet was lysed in ice-cold 1× RIPA buffer by sonication (Bioruptor, 30 s on, 30 s off, 3 cycles, 4 °C) and shaking at 2,000 rpm at 4 °C for 15 min. After the final centrifugation for 10 min at 10,000*g* (mouse) or 2,000*g* (human) at 4 °C, the RIPA soluble and insoluble fractions were separated and stored at −80 °C. Throughout the isolation, siliconized (Sigmacote, Sigma-Aldrich) or Protein LoBind tubes (Eppendorf) were used to increase recovery of vessels.

### ELISA

MFG-E8 protein levels in mouse and human samples were measured by commercial ELISA (R&D Systems) according to the manufacturer’s instructions. Mouse brain homogenates (10%) were pre-diluted 1:5 and RIPA-soluble vessel fractions 1:4 and 1:10. For human samples, brain RIPA homogenates (5%) were pre-diluted 1:20 and RIPA-soluble vessel fractions 1:100. MFG-E8 levels were normalized to the total protein content as measured by BCA protein assay (Pierce). Measurements were performed on a FLUOstar Omega reader (BMG Labtech, MARS v. 2.4).

Quantification of human amyloid-β in patients with Alzheimer’s disease and transgenic mice was performed by human amyloid-β V-plex assay (6E10 or 4G8; Meso Scale Discovery, Workbench 3.0) or by the SIMOA Human Aβ42 2.0 Kit (Quanterix) in FA-extracted samples (brain homogenates, isolated cerebral vessels or 50,000 isolated microglia) according to the manufacturer’s instructions^52,57^. Murine and human samples were pre-diluted in a range of undiluted to 1:100 (human and murine) for brain homogenates and undiluted to 1:3,000 (human) or 1:2 to 1:1,000 (murine) for vessel extracts (soluble and insoluble) in order to measure amyloid-β concentration in the linear range of the standard curve. Murine amyloid-β was measured by amyloid-β triplex assay (4G8, Meso Scale Discovery) in diethylamine (DEA)-extracted brain homogenates (see ‘Protein extraction’).

Cytokines of microglial cells (50,000 cells in 50 mM Tris pH 8, 150 mM NaCl, 5 mM EDTA) were measured using the mouse pro-inflammatory panel 1 V-plex plate (Meso Scale Discovery) according to the manufacturer’s protocol.

### Western blotting

Samples were diluted and denatured in loading buffer (10% glycerol, 2% SDS, 2% β-mercaptoethanol or 100 mM DTT, 0.1 M Tris-HCl pH 8.6), sonicated (3× 5 s for 6E10), heated to 95 °C for 5 min and loaded on a Bis-Tris 4–12% or Tris-Tricine 10–20% gradient gel (NuPage, Invitrogen). After electrophoresis in Tricine or MES SDS Running Buffer (NuPage, Invitrogen), gels were transferred to a nitrocellulose membrane in a semi-dry blotting system (200 mA, 45–75 min). Transfer was confirmed by Ponceau-S staining. For detection of amyloid-β and medin/MFG-E8 (or fragments), membranes were boiled in PBS for 5 min at 90 °C. For membrane stripping and improved detection of human medin, membranes were incubated 3× 7 min in pre-heated 100 mM glycine (pH 2) buffer. Blocking was performed either with 5% milk (6E10, GAPDH, 6B3, SMA, β-actin; PDGFR-β in 5% milk-TBST) or 5% donkey serum (polyclonal anti-murine MFG-E8) in PBST (PBS + 0.05% Tween) for 1 h. Subsequently, membranes were incubated overnight at 4 °C with the primary antibody in PBST. Primary antibodies used were goat polyclonal anti-murine MFG-E8 (R&D systems, 1:1,000), anti-human MFG-E8 (R&D Systems, 1:1,000), anti-human medin 6B3 (Prothena Biosciences Limited, 1:2,500 in 5% BSA-PBST), anti-GAPDH (Acris Antibodies GmbH, 1:100,000), anti-amyloid-β 6E10 (BioLegend, 1:2,500), anti-α-SMA (1A4, Dako, 1:1,000), anti-β-actin (Abcam, 1:2,500), anti-PDGFR-β (Cell Signaling, 1:1,000). Membranes were then probed with the respective secondary horseradish peroxidase (HRP)-labelled antibodies (1:20,000, Jackson ImmunoLaboratories). Protein bands were detected using chemiluminescent peroxidase substrate (Super Signal West Pico Plus or Dura, Thermo Fisher Scientific). Densitometric values of single protein bands (amyloid-β, APP, CTF-β, GAPDH, α-SMA, β-actin; PDGFR-β, full-length MFG-E8) or fragments or aggregates (6B3, 1–80 kDa) were analysed with the software package Aida (Stella 3200, Raytest) or Fiji/ImageJ (v. 2.3) and normalized to GAPDH or β-actin.

### Medin depletion of aortic extracts

Medin depletion was performed similarly to as described^38^. In brief, 200 μl of paramagnetic beads coated with Protein G (Dynabeads) were washed 3 times in sterile PBS+0.02% Tween and incubated overnight with 1 ml tissue-culture supernatant of the monoclonal anti-human medin 1H4 antibody. Aortic extract was pre-diluted 1:10 in sterile PBS and 100 μl of the diluted extract were incubated for 2 h with one quarter of the 1H4-Protein G-Dynabeads-complex. This step was then repeated three times, with the final incubation taking place overnight at 4 °C. The final supernatant was used for injection. Paramagnetic beads from each step were washed 3 times with PBS and subjected to elution in loading buffer. Final supernatant and eluted bead material from each step were collected for Western blotting using 6B3 as detection antibody.

### In vivo inoculations

Medin aggregates were purified from fresh-frozen 100 mg human aorta (*n* = 2 individuals, 2 extractions per individual were pooled before injection) and amyloid-β was purified from the brain of one 28-month-old APP23 transgenic mouse, as previously described^3^. Quantification of amyloid-β in the human aorta extracts was performed after FA extraction using the MesoScale Discovery platform (see section ‘ELISA’), yielding no detectable signals. For mouse aorta seeding extracts, aortas from aged *Mfge8* C2 KO and wild-type mice were homogenized in sterile PBS (10 mg tissue per 100 µl) and sonicated three times for 5 s (LabSonic); samples were adjusted to the same total protein concentration (2 µg µl^−1^).

Intrahippocampal injections (2.5 µl per hippocampus) were done bilaterally in pre-depositing 2- to 4-month-old female and male APP23 mice^14^. Whenever possible, age-matched littermates were used for injections to exclude any contribution of endogenous amyloid-β deposits to the observed seeding effects (note that hippocampal deposits are exceedingly rare in 9–10 months old APP23 mice, and can also be distinguished from seeded amyloid-β aggregates due to their characteristic induction patterns). After anaesthesia with ketamine/xylazine (100 mg kg^−1^ to 10 mg kg^−1^ of body weight), hippocampal injections (anteroposterior, −2.5 mm; left/right, ±2.0 mm; dorsoventral, −1.8 mm) were delivered with a Hamilton syringe^58^ at a speed of 1.25 μl min^−1^. The syringe was kept in place for an additional 2 min and then slowly withdrawn. The surgical incision was closed, and the mice were closely monitored until regaining consciousness. After 6 months of incubation, the mice were sacrificed and the brains processed for histologic staining with anti-amyloid-β antibody (CN6^59^, 1:1,000) and Congo Red. Amyloid-β load was quantified stereologically (as described^60^; see also ‘Image analysis’).

### Histology and immunostaining

Paraffin sections were deparaffinized and rehydrated using standard protocols. Free-floating brain sections were washed in PBS and endogenous peroxidase was quenched by incubation of the sections with 0.3% hydrogen peroxide (AppliChem) in PBS for 30 min. For staining of human brain tissue, sections were pre-treated with 1 µg ml^−1^ proteinase K (in 1 mM CaCl_2_, 50 mM Tris buffer, pH 7.6) at 37 °C for 30 min, followed by heat deactivation in 10 mM EDTA (pH 6) at 90 °C for 10 min (refs. ^3,61^). Human aorta paraffin sections were boiled in citrate buffer (1.8 mM citric acid, 8.2 mM trisodium citrate, pH 6) at 90 °C for 30 min. Nonspecific antibody binding was blocked by incubation with 5% normal serum of the secondary antibody species (in 0.3% Triton X-100 in PBS), and primary antibody was incubated at 4 °C over 1 or 2 nights, followed by washing and incubation with the secondary antibody (diluted in 1% serum-PBS) using either ABC and Peroxidase Substrate kits (Vectastain) or appropriate fluorescently labelled secondary antibodies (according to the manufacturer’s instructions, Invitrogen or Jackson ImmunoResearch, 1:250 and Biolegend BV421, 1:100). To reduce autofluorescence (from various sources such as lipofuscin, elastin or collagen) in human brain sections, TrueBlack Quencher (Biotium) was applied (1:20 in 70% ethanol) for 5–10 s, according to the manufacturer’s instructions.

Primary antibodies used were anti-human medin antibody (clone 1H4 hybridoma supernatant, 1:2), goat polyclonal anti-mouse MFG-E8 antibody (R&D Systems, 1:1,000), anti-IBA1 (WAKO, 1:1,000), anti-PU.1 (Cell Signaling, 1:1,000 for immunohistochemistry, 1:250 for immunofluorescence), anti-ALDH1L1 (Abcam, 1:100), anti-human MFG-E8 (R&D Systems, 1:500), anti-SMA (Abcam, 1:200), anti-APP A4 (Millipore, 1:1,000), anti-amyloid-β (CN6^59^, 1:1,000), anti-vimentin (Abcam, 1:250), anti-murine serpin A3N (R&D Systems, 1:200), anti-GFAP (Biozol, 1:500). Amyloid staining was performed using Methoxy-X04 (0.4% vol of 10 mg ml^−1^ in DMSO and 0.8% vol CremophorEL in PBS) for 15 min at room temperature, Congo red or LCO staining (2.4 µM qFTAA and 0.77 µM hFTAA in PBS^58^), according to standard protocols. Prussian blue staining was used to visualize cerebral microbleeds by staining ferric iron in hemosiderin as described^19^.

### Image analysis

Brightfield images were acquired using a Zeiss Axioplan 2 with the AxioVision 4.7 software (Zeiss) using a 4×/0.10 or 40×/0.75 objective, with fixed camera exposure time and lamp intensity for comparative stainings. Optical sections of fluorescent stainings were acquired with an air 20×/0.5 NA or an oil immersion 40×/1.3 NA or 63×/1.4 NA objective either on a Zeiss LSM 510 META (Axiovert 200M; LSM software 4.2, Carl Zeiss), Zeiss LSM 700 (ZEN 2012 SP5), or Leica TCS SP8 X (LAS X, Leica) confocal microscope using sequential excitation of fluorophores (BV421, A488, A568, A555 and A647). Maximum intensity projections were generated with Zeiss, Fiji or Imaris software. All imaging and analysis steps were performed by a blinded observer.

Amyloid-β (compact and diffuse, CAA) load was determined based on Congo red and anti-amyloid-β staining using the area fraction fractionator technique^38^. Stereological analysis was performed by a blinded observer on sets of every 36th systematically sampled brain sections throughout the neocortex (for quantification of cortical amyloid-β load in the 2- to 4-month-old APPPS1 × *Mfge8* or in the 9- to 12-month-old APP23 x *Mfge8* mice, respectively) or in a set of every 12th sampled section for quantification of hippocampal seeding induction using an Axioskop microscope (Zeiss) equipped with a motorized *x-y-z* stage coupled to a video-microscopy system (Microfire Optronics). Analysis was conducted using the Stereo Investigator 6 software (MBF Bioscience). Amyloid-β load was calculated as area (%) covered by Congo red and anti-amyloid-β staining. Since the 6-month-old female APP23 x *Mfge8* mice only had 1–8 plaques per set of every 12th section, plaque number was quantified by blinded counting of the number of plaques rather than stereological analysis. Male 6-month-old APP23 × *Mfge8* WT or APP23 × *Mfge8* C2 KO mice had not developed plaques yet and were therefore not included in this analysis. For ease of visual comparison, stereological counts were normalized to the mean value of the *Mfge8* wild-type mice for each age-group.

Frequency of CAA (positive for amyloid-β and Congo Red) and the number of hemosiderin-positive microhaemorrhages was manually assessed throughout the region of interest (every 36th section in the cortex for CAA and every 12th section for hemosiderin; and every 12th section of the hippocampus, striatum and thalamus, according to previous descriptions^19^).

Co-localization of MFG-E8 or medin (1H4) with amyloid-β and amyloid (Methoxy-X04) was analysed on 10-µm-thick *z*-stacks (imaged at 40× magnification) of ~30 cortical plaques and ~25 amyloid-β-laden blood vessels per mouse or ~20–30 amyloid-laden vessels per individual (on average 17 leptomeningeal and 9 parenchymal SMA-positive vessels in the frontal and occipital cortex). To quantify the percentage of co-localization, images were processed with Imaris (Bitplane, v. 9.7.2), and 3D surfaces were created for each channel based on a fixed intensity threshold. The overlap volume between two surfaces (for example, MFG-E8 and medin, and amyloid-β in CAA-affected vessels) was measured using the inbuilt function Overlapped Volume to Surfaces Surfaces. The percentage of co-localization was calculated as the ratio of overlapped surface volume to total surface volume.

For analysis of LCO staining, 8 µm-thick *z*-stacks of plaques were acquired using sequential excitation of the A647 (IBA1) or BV421 fluorophore (APP) and LCOs at 40× magnification. qFTAA and hFTAA were excited with the 405 nm laser. Maximum intensity projections of the images were semi-automatically analysed with a custom macro in Fiji, which performed the following functions: after background removal using a rolling background subtraction filter of 100 pixels size, selection of plaques as regions of interest, splitting of fluorescence channels, generation of maximum intensity projections, and application of fixed intensity thresholds. For every plaque, plaque size and the area of the different stainings within the region of interest were then determined based on thresholded values. For each individual plaque, the area of qFTAA and the area of hFTAA staining was quantified and expressed as a ratio of the qFTAA to hFTAA area to derive a measure that is independent of overall plaque size. After calculating this ratio for each plaque, the mean value for all plaques per mouse was calculated.

The numbers of microglia and astrocytes per brain were stereologically quantified by counting IBA1-positive (microglial), ALDH1L1-positive (homeostatic, astrocytic) or GFAP-positive (activated, astrocytic) cells using the optical fractionator technique (at 40× magnification; Stereo Investigator 6, MBF Bioscience, with three-dimensional dissectors), as previously described^52,60^. The numbers of plaque-associated PU.1-positive microglia and GFAP-positive astrocytes were normalized to Congo Red-positive area for every individual plaque. Microglial nuclei were exclusively counted in the two-fold radius of plaque diameter. GFAP-positive cell area was calculated by measuring the radius of GFAP-positive cell staining per plaque. For further detailed analysis of disease-associated astrocytes, four cortical regions of interest per mouse were chosen and 25 µm-thick *z-*stacks were acquired (using sequential excitation of the fluorophores A488, A555, A647 and Methoxy-X04, at 40× magnification). For data analysis, projections of the images were analysed using Fiji software. Mean fluorescence of the vimentin and serpin A3N signal in astrocytes was measured based on GFAP-positive area selection by the threshold function.

### Electron microscopy of tissue sections

For electron microscopy, mice were perfused with PBS, followed by a mixture of 4% PFA and 0.5% glutaraldehyde in 0.1 M cacodylate buffer (pH 7.4, Science Services) for 15 min. Serial frontal brain sections were cut with a vibratome (Leica VT1000S), washed in TBS, incubated in 0.1% NaBH4 (Sigma-Aldrich), and blocked with 5% BSA for 1 h at room temperature to reduce nonspecific staining. For MFG-E8 staining, goat polyclonal anti-mouse MFG-E8 (R&D systems, 1:1,000) was used as primary antibody followed by a biotinylated specific anti-IgG (Vector Laboratories, 1:200) as secondary antibody. After washing in TBS, sections were incubated in avidin–biotin–peroxidase complex (ABC-Elite; Vector Laboratories) for 90 min at room temperature and were reacted with diaminobenzidine (DAB) solution (Vector Laboratories) at room temperature. Sections were silver-intensified by incubation in 3% hexamethylenetetramine (Sigma-Aldrich), 5% silver nitrate (AppliChem), and 2.5% disodium tetraborate (Sigma-Aldrich) for 10 min at 60 °C, in 1% tetrachlorogold solution (AppliChem) for 3 min, and in 2.5% sodium thiosulfate (Sigma-Aldrich) for 3 min. After staining, sections were washed in 0.1 M cacodylate buffer, osmicated (0.5% OsO4 in cacodylate buffer), dehydrated (70% ethanol containing 1% uranyl acetate (Serva)), and embedded in Durcupan (Sigma-Aldrich). Ultrathin sections were collected on single-slot Formvar-coated copper grids that were contrast-enhanced with lead citrate for 4 min and examined using a Zeiss EM 900 electron microscope.

### ThT fluorescence aggregation assay, and electron microscopy of recombinant medin or amyloid-β co-aggregation and human aorta extracts

ThT fluorescence assays were carried out on a Flexstation 3 microplate reader (Molecular Devices). Experiments were carried out in sealed 96-well, black-walled, clear-bottomed microplates (Nunc). Data were recorded every 5 min using bottom read mode, with excitation at 440 nm and emission at 490 nm. The assay was carried out using 15 µM medin (produced recombinantly as previously described^62^) or 20 µM Aβ40 (BioLegend) alone or co-incubated in 50 mM Tris, 150 mM NaCl, 5 mM EDTA, pH 8 with 2 µM ThT at 37 °C under quiescent conditions with 5 s shaking before each reading. For immuno-electron microscopy of recombinant fibrils, peptides were aggregated by incubating medin and Aβ40 alone or together under the same conditions as used for ThT analysis at 37 °C for 7 days. Four-microlitre aliquots of fibrils were loaded onto carbon-coated copper grids and left for 2 min, the excess was removed by filter paper, and blocked in 1:10 goat serum in PBS, pH 8.2, containing 1% BSA, 500 ml per litre Tween-20, 10 mM Na EDTA, and 0.2 g l^−1^ NaN_3_, for 15 min. Grids were then incubated with 1:500 medin antibody (custom antibody from GenicBio Hong Kong) and amyloid-β antibody (6E10, BioLegend) for 2 h at room temperature, rinsed in 3× 2 min PBS+, and then immunolabelled using 6 nm gold particle-conjugated goat anti-rabbit and 14 nm donkey anti-mouse IgG secondary probe (1:50) for 1 h at room temperature. After five 2-min PBS+ and five 2-min distilled water rinses, the grids were negatively stained using 4% uranyl acetate for 30 s. Samples were visualized on a Tecnai 10 electron microscope at 100 kV. For staining of medin aggregates isolated from human aortas (as described under ‘In vivo inoculations’), the same protocol was used for immuno-electron microscopy, with slight modifications, namely using the 1H4 antibody to stain medin aggregates and a 1:10 dilution of 10 nm gold particle-conjugated goat anti-rat IgG secondary probe.

### Synthesis of CT-medin peptide and Met-Aβ and thioflavin T co-aggregation assays

Met-Aβ was produced in-house as previously described^63^ using the human Met-Aβ1–42 expression plasmid, provided by C. Gomes. The C-terminal medin fragment (CT-medin: EVTGIITQGARNFGSVQFVASYK) was synthesized using an Intavis Multipep RSi solid phase peptide synthesis robot. Peptide preparations were stored as precipitates (−20 °C) and purity (>90%) was evaluated using RP-HPLC purification protocols. Peptide stocks were prepared by dissolving in appropriate buffer and filtering through 0.2-μm spin-down filters (Millipore, Germany).

ThT fluorescence assays were carried out on an Fluostar Omega microplate reader (BMG Labtech, Germany). Experiments were carried out in sealed 96-well, half-area flat clear-bottomed microplates (Corning). Data were recorded every 10 min using bottom read mode, with excitation at 440 nm and emission at 490 nm. Prior to ThT assay experiments, lyophilised samples of Met-Aβ were suspended for 1 h at ambient conditions in 7 M guanidinium chloride in 50 mM Tris (pH 8), then centrifuged (15,000 rpm at 4 °C) for 5 min. The resulting supernatant was subsequently injected in a Superdex 75 10/300 GL gel filtration column (GE Healthcare), following equilibration with 50 mM Tris buffer (pH 8). The eluted monomeric fraction was isolated and kept on ice, while the concentration of the eluent was determined using a NanoDrop 2000 (Thermo Fisher Scientific), using a molecular weight of 4,645 Da and an extinction coefficient of 1.49. The peptide was diluted to a final concentration of 10μM just prior to plating. Similarly, CT-medin was dissolved in 50 mM Tris (pH 8) at a concentration of 10 μM. ThT co-aggregation and seeding assays (25 μM ThT) were run at 30 °C under quiescent conditions with 5 s shaking before each reading. For seeding assays, seeds of Met-Aβ and CT-medin were prepared by aggregating both peptides using the same protocol harvesting end-state aggregates from the plates in the absence of ThT. The end-state amyloid fibrils were sonicated for 15 min (30 s on, 30 s off) at 10 °C, using a Bioruptor Pico sonication device (Diagenode) and 0.5 µM were added to the monomeric peptides.

Determination of half-times was performed by normalizing and fitting the derived ThT curves using:

$$y={y}_{0}+\frac{{y}_{\max }-{y}_{0}}{1+\exp (-(x-{t}_{1/2}))\times k}$$

where fluorescence intensity (*y*) is represented as a function of time (*x*). *y*_max_ and *y*_0_ indicate maximum and starting fluorescence values, respectively, whereas *t*_1/2_ and *k* are the kinetic half-times and elongation rates of the fitted curves, respectively. *t*_1/2_ values were determined separately for each individual replicate per sample.

For the analysis of LCO spectra of co-aggregated peptides, end-state aggregates were prepared by running parallel assay plates in the absence of ThT as described above. Independent peptide preparations (*n* = 3) were split into three equal aliquots before incubation and then run in triplicates, resulting in 27 total replicates. Suspensions (20 μl) of each peptide replicate were then mixed with pFTAA (0.5 μM) and fluorescence emission spectra (465 nm–600 nm) were subsequently recorded in low-volume 384-well black plates with clear bottom (Corning) by exciting at 440 nm, using a ClarioStar plate reader at 30 °C (BMG Labtech, Germany). Spectral acquisitions were background-subtracted and analysed using Prism 9.

### Sequence alignment and structural modelling

Sequence alignment of medin and Aβ42 was performed using Clustal Omega^64^. Conservation track and colour-coding based on the Blosum62 substitution matrix was applied from Jalview^65^. A continuous 13 amino acid-long C-terminal peptide of medin of with high homology to the C-terminus of amyloid-β and containing the most aggregation-prone region of medin^3^ was chosen to examine the interaction of medin with four recently reported amyloid-β assemblies. Aβ amyloid fibril structures extracted from Alzheimer’s disease brains and composed of Aβ42 (PDB ID: 7Q4B and 7Q4M), Aβ40 (PDB ID: 6W0O) or a mixture of different amyloid-β peptides isolated from the vasculature (CAA; PDB ID: 6SHS) were energetically optimized using the RepairPDB function available in the FoldX force field^66^. Successively, the N-terminal residues (up to A30) from a single protofilament were removed from each template structure. Finally, to assess structural compatibility, we threaded query sequences using as template the central buried rung of the derived amyloid-β template structures. Calculated differentials in free energy between the derived modelled structures and the starting template were used as an indicator of structural fitting between the query sequences and each corresponding amyloid-β fibril fold (ΔΔ*G* values).

### Statistics and reproducibility

Statistical analysis was performed using Prism 9 software. Data were tested for normal distribution (Shapiro–Wilk test) and statistical outliers were identified and removed (ROUT method), where necessary. If data were normally distributed, one- or two-way ANOVAs were performed, followed by Tukey’s multiple comparison test. Because we could not detect overt sex effects in our data sets, we did not consider sex as an independent variable in our analyses. If data were not normally distributed, a non-parametric test (Kruskal–Wallis) was performed, followed (if *P* < 0.05) by multiple comparison of the mean ranks with Dunn’s correction. For pairwise comparisons, non-parametric Mann–Whitney tests were used; all tests were two-tailed, with the exception of data on LCO ratios in Fig. 3b, where the hypothesis that the ratio would be shifted towards higher values (that is, more compact or fibrillar amyloid) in *Mfge8* C2 KO mice was posited a priori based on immuno-electron microscopy data in Fig. 3a.

Linear regressions were performed using JMP software (version 14.2.0 or higher). If necessary, data were first log_10_-transformed to achieve a normal distribution. Data were then analysed using the ‘fit model’ function, generating parameter estimates as well as residual versus leverage plots, where a least-squares line (red) and confidence bands (shaded red) provide a visual representation of the statistical significance (at the 5% level) of the effect of *x*; a significant effect is evident by the crossing of the confidence lines (shaded red/red) through the blue line in the graph, which indicates the mean of the *y* leverage residuals. To calculate the data points in the graph, the mean value of *y* is added to the *y* residuals and the mean of the *x* value is added to the *x* residuals, generating ‘leverage residuals’, and these pairs of residuals are then used to generate the effect leverage plots shown^67^.

Micrographs shown in the figures were selected for being representative of the general staining pattern—all immunostainings were reproduced at least twice (often multiple times), with equivalent results. Similarly, all western blots were reproduced at least twice, with equivalent results.

### Reporting summary

Further information on research design is available in the Nature Portfolio Reporting Summary linked to this article.

## Online content

Any methods, additional references, Nature Portfolio reporting summaries, source data, extended data, supplementary information, acknowledgements, peer review information; details of author contributions and competing interests; and statements of data and code availability are available at 10.1038/s41586-022-05440-3.

### Supplementary information

Supplementary Figures This file contains uncropped immunoblots for Figs. 1h, 2d,g and 4b,d and Extended Data Figs. 2b,d and 5b.

Reporting Summary

Supplementary Table 1 Human tissue information. Patient and tissue information for histological and biochemical analyses performed in this study.

Supplementary Table 2 ROSMAP patient information and RNA-sequencing data. Patient information and RNA-sequencing values for ROSMAP patient data analysed in this study.

## Data Availability

Reagents and any further information are available on request to the corresponding author. Source data are provided with this paper.

## Source data

Source Data Figs. 1–4 and Source Data Extended Data Figs. 2–6

## References

[1] Häggqvist B (1999). Medin: an integral fragment of aortic smooth muscle cell-produced lactadherin forms the most common human amyloid. Proc. Natl Acad. Sci. USA.

[2] Mucchiano G, Cornwell GG, Westermark P (1992). Senile aortic amyloid. Evidence for two distinct forms of localized deposits. Am. J. Pathol..

[3] Degenhardt K (2020). Medin aggregation causes cerebrovascular dysfunction in aging wild-type mice. Proc. Natl Acad. Sci. USA.

[4] Benson MD (2020). Amyloid nomenclature 2020: update and recommendations by the International Society of Amyloidosis (ISA) nomenclature committee. Amyloid.

[5] Eisenberg D, Jucker M (2012). The amyloid state of proteins in human diseases. Cell.

[6] Iadanza MG, Jackson MP, Hewitt EW, Ranson NA, Radford SE (2018). A new era for understanding amyloid structures and disease. Nat. Rev. Mol. Cell Biol..

[7] Peng S, Glennert J, Westermark P (2005). Medin-amyloid: a recently characterized age-associated arterial amyloid form affects mainly arteries in the upper part of the body. Amyloid.

[8] Karamanova N (2020). Endothelial immune activation by medin: potential role in cerebrovascular disease and reversal by monosialoganglioside-containing nanoliposomes. J. Am. Heart Assoc..

[9] Migrino RQ (2020). Cerebrovascular medin is associated with Alzheimer’s disease and vascular dementia. Alzheimers Dement..

[10] Davies HA (2019). Idiopathic degenerative thoracic aneurysms are associated with increased aortic medial amyloid. Amyloid.

[11] Peng S (2007). Role of aggregated medin in the pathogenesis of thoracic aortic aneurysm and dissection. Lab Invest..

[12] Peng S (2002). Medin and medin-amyloid in ageing inflamed and non-inflamed temporal arteries. J. Pathol..

[13] Radde R (2006). Aβ42-driven cerebral amyloidosis in transgenic mice reveals early and robust pathology. EMBO Rep..

[14] Sturchler-Pierrat C (1997). Two amyloid precursor protein transgenic mouse models with Alzheimer disease-like pathology. Proc. Natl Acad. Sci. USA.

[15] Silvestre J-S (2005). Lactadherin promotes VEGF-dependent neovascularization. Nat. Med..

[16] Calhoun ME (1999). Neuronal overexpression of mutant amyloid precursor protein results in prominent deposition of cerebrovascular amyloid. Proc. Natl Acad. Sci. USA.

[17] Herzig MC (2004). Abeta is targeted to the vasculature in a mouse model of hereditary cerebral hemorrhage with amyloidosis. Nat. Neurosci..

[18] Schmidt ML, Robinson KA, Lee VM, Trojanowski JQ (1995). Chemical and immunological heterogeneity of fibrillar amyloid in plaques of Alzheimer’s disease and Down’s syndrome brains revealed by confocal microscopy. Am. J. Pathol..

[19] Winkler DT (2001). Spontaneous hemorrhagic stroke in a mouse model of cerebral amyloid angiopathy. J. Neurosci..

[20] Suzuki N (1994). High tissue content of soluble β1–40 is linked to cerebral amyloid angiopathy. Am. J. Pathol..

[21] Greenberg SM (2020). Cerebral amyloid angiopathy and Alzheimer disease—one peptide, two pathways. Nat. Rev. Neurol..

[22] Marazuela P (2021). MFG-E8 (LACTADHERIN): a novel marker associated with cerebral amyloid angiopathy. Acta Neuropathol. Commun..

[23] Mostafavi S (2018). A molecular network of the aging human brain provides insights into the pathology and cognitive decline of Alzheimer’s disease. Nat. Neurosci.

[24] De Jager PL (2018). A multi-omic atlas of the human frontal cortex for aging and Alzheimer’s disease research. Sci. Data.

[25] Blauwendraat C (2016). Comprehensive promoter level expression quantitative trait loci analysis of the human frontal lobe. Genome Med..

[26] Aibar S (2017). SCENIC: single-cell regulatory network inference and clustering. Nat. Methods.

[27] Skene NG, Grant SGN (2016). Identification of vulnerable cell types in major brain disorders using single cell transcriptomes and expression weighted cell type enrichment. Front. Neurosci..

[28] Wegenast-Braun BM (2012). Spectral discrimination of cerebral amyloid lesions after peripheral application of luminescent conjugated oligothiophenes. Am. J. Pathol..

[29] Nyström S (2013). Evidence for age-dependent in vivo conformational rearrangement within Aβ amyloid deposits. ACS Chem. Biol..

[30] Yuan P (2016). TREM2 haplodeficiency in mice and humans impairs the microglia barrier function leading to decreased amyloid compaction and severe axonal dystrophy. Neuron.

[31] Brown GC, Neher JJ (2014). Microglial phagocytosis of live neurons. Nat. Rev. Neurosci..

[32] Yang Y (2022). Cryo-EM structures of amyloid-β 42 filaments from human brains. Science.

[33] Ghosh U, Thurber KR, Yau W-M, Tycko R (2021). Molecular structure of a prevalent amyloid-β fibril polymorph from Alzheimer’s disease brain tissue. Proc. Natl Acad. Sci. USA.

[34] Kollmer M (2019). Cryo-EM structure and polymorphism of Aβ amyloid fibrils purified from Alzheimer’s brain tissue. Nat. Commun..

[35] Li H (2015). Apolipoprotein D modulates amyloid pathology in APP/PS1 Alzheimer’s disease mice. Neurobiol. Aging.

[36] Kuiperij HB (2020). Apolipoprotein D: a potential biomarker for cerebral amyloid angiopathy. Neuropathol Appl Neurobiol.

[37] Pedrero-Prieto CM (2020). A comprehensive systematic review of CSF proteins and peptides that define Alzheimer’s disease. Clin. Proteomics.

[38] Meyer-Luehmann M (2006). Exogenous induction of cerebral beta-amyloidogenesis is governed by agent and host. Science.

[39] Fritschi SK (2014). Highly potent soluble amyloid-β seeds in human Alzheimer brain but not cerebrospinal fluid. Brain.

[40] Kokjohn TA (2011). Chemical characterization of pro-inflammatory amyloid-beta peptides in human atherosclerotic lesions and platelets. Biochim. Biophys. Acta.

[41] Roher AE (2009). Amyloid beta peptides in human plasma and tissues and their significance for Alzheimer’s disease. Alzheimers Dement..

[42] Davies HA, Madine J, Middleton DA (2015). Comparisons with amyloid-β reveal an aspartate residue that stabilizes fibrils of the aortic amyloid peptide medin. J. Biol. Chem..

[43] Larsson A (2007). Unwinding fibril formation of medin, the peptide of the most common form of human amyloid. Biochem. Biophys. Res. Commun..

[44] Nelson CP (2017). Association analyses based on false discovery rate implicate new loci for coronary artery disease. Nat. Genet..

[45] Nikpay M (2015). A comprehensive 1,000 Genomes-based genome-wide association meta-analysis of coronary artery disease. Nat. Genet..

[46] van der Harst P, Verweij N (2018). Identification of 64 novel genetic loci provides an expanded view on the genetic architecture of coronary artery disease. Circ. Res..

[47] Soubeyrand S (2019). Regulation of MFGE8 by the intergenic coronary artery disease locus on 15q26.1. Atherosclerosis.

[48] Iturria-Medina Y (2016). Early role of vascular dysregulation on late-onset Alzheimer’s disease based on multifactorial data-driven analysis. Nat. Commun..

[49] Rabin JS (2018). Interactive associations of vascular risk and β-amyloid burden with cognitive decline in clinically normal elderly individuals: findings from the Harvard Aging Brain Study. JAMA Neurol..

[50] Boyle PA (2015). Cerebral amyloid angiopathy and cognitive outcomes in community-based older persons. Neurology.

[51] Uhlmann RE (2020). Acute targeting of pre-amyloid seeds in transgenic mice reduces Alzheimer-like pathology later in life. Nat. Neurosci..

[52] Wendeln A-C (2018). Innate immune memory in the brain shapes neurological disease hallmarks. Nature.

[53] Lee Y-K, Uchida H, Smith H, Ito A, Sanchez T (2019). The isolation and molecular characterization of cerebral microvessels. Nat. Protoc..

[54] Boulay A-C, Saubaméa B, Declèves X, Cohen-Salmon M (2015). Purification of mouse brain vessels. J. Vis. Exp..

[55] Matthes F (2021). An improved method for physical separation of cerebral vasculature and parenchyma enables detection of blood–brain-barrier dysfunction. NeuroSci.

[56] Bourassa P, Tremblay C, Schneider JA, Bennett DA, Calon F (2019). Beta-amyloid pathology in human brain microvessel extracts from the parietal cortex: relation with cerebral amyloid angiopathy and Alzheimer’s disease. Acta Neuropathol..

[57] Ye L (2017). Aβ seeding potency peaks in the early stages of cerebral β-amyloidosis. EMBO Rep..

[58] Rasmussen J (2017). Amyloid polymorphisms constitute distinct clouds of conformational variants in different etiological subtypes of Alzheimer’s disease. Proc. Natl Acad. Sci. USA.

[59] Eisele YS (2010). Peripherally applied Aβ-containing inoculates induce cerebral β-amyloidosis. Science.

[60] Varvel NH (2015). Replacement of brain-resident myeloid cells does not alter cerebral amyloid-β deposition in mouse models of Alzheimer’s disease. J. Exp. Med..

[61] Kai H (2012). Enhanced antigen retrieval of amyloid β immunohistochemistry: re-evaluation of amyloid β pathology in Alzheimer disease and its mouse model. J. Histochem. Cytochem..

[62] Davies HA (2015). Oxidative stress alters the morphology and toxicity of aortic medial amyloid. Biophys. J..

[63] Konstantoulea K (2022). Heterotypic amyloid β interactions facilitate amyloid assembly and modify amyloid structure. EMBO J..

[64] Sievers F (2011). Fast, scalable generation of high-quality protein multiple sequence alignments using Clustal Omega. Mol. Syst. Biol..

[65] Waterhouse AM, Procter JB, Martin DMA, Clamp M, Barton GJ (2009). Jalview Version 2-a multiple sequence alignment editor and analysis workbench. Bioinformatics.

[66] Schymkowitz J (2005). The FoldX web server: an online force field. Nucleic Acids Res..

[67] Sall J (1990). Leverage plots for general linear hypotheses. Am. Stat..

[68] Habib N (2020). Disease-associated astrocytes in Alzheimer’s disease and aging. Nat. Neurosci..

[69] Singh M, Bansal V, Feschotte C (2020). A single-cell RNA expression map of human coronavirus entry factors. Cell Rep..

[70] Mathys H (2019). Single-cell transcriptomic analysis of Alzheimer’s disease. Nature.

[71] Yang AC (2022). A human brain vascular atlas reveals diverse mediators of Alzheimer’s risk. Nature.

[72] Ximerakis M (2019). Single-cell transcriptomic profiling of the aging mouse brain. Nat. Neurosci..

[73] He L (2018). Single-cell RNA sequencing of mouse brain and lung vascular and vessel-associated cell types. Sci. Data.

[74] Vanlandewijck M (2018). A molecular atlas of cell types and zonation in the brain vasculature. Nature.

