# Supplementary material for: Medin co-aggregates with vascular amyloid-β in Alzheimer’s disease
Source: Nature. 2022 Nov 16;612(7938):123–31. doi: 10.1038/s41586-022-05440-3 (PMC9712113; doi:10.1038/s41586-022-05440-3)
Supplement: Supplementary file 1 — This file contains uncropped immunoblots for Figs. 1h, 2d,g and 4b,d and Extended Data Figs. 2b,d and 5b. [file 41586_2022_5440_MOESM1_ESM.pdf]

nature portfolio

https://doi.org/10.1038/s41586-022-05440-3

Supplementary information

Medin co-aggregates with vascular amyloid-β in Alzheimer's disease

In the format provided by the authors and unedited

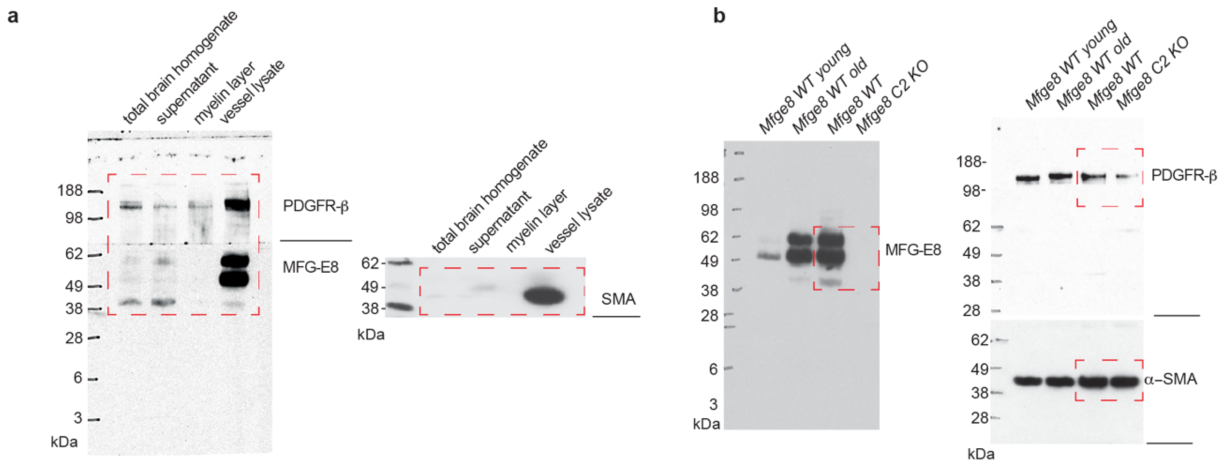

SI Figure 1 | Uncropped Western Blots from main Figure 1h.
Red rectangles indicate the sections displayed in Fig.1h. **a**, Western Blots of total brain homogenate, supernatant, myelin layer and vessel lysate of a *Mfge8* WT animal. Following transfer, the membrane was cut into different molecular weight sections (cutting locations are indicated with horizontal black lines) to detect MFG-E8 and PDGFR-β (*left*). Following detection of MFG-E8 (a), the membrane was stripped to remove antibodies (see Methods for details), the membrane was cut again and re-probed with an antibody against α-SMA (clone 1A4) (*right*). **b**, Western Blot of RIPA-soluble vessel lysate from an *Mfge8* WT and *Mfge8* C2 KO animal. After detection of MFG-E8, the membrane was stripped and cut into different molecular weight sections for detection of PDGFR-β and α-SMA. β-actin was run as a loading control on the same gel.

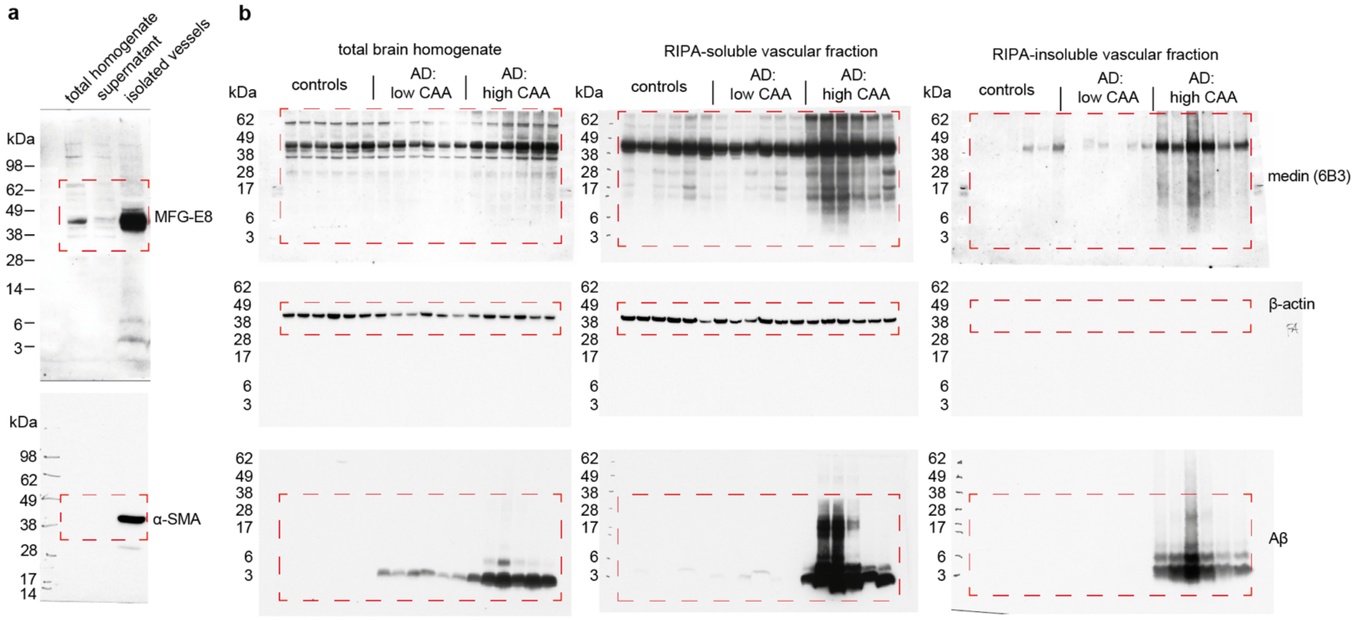

SI Figure 2 | Uncropped Western Blots from main Figure 2.
Red rectangles indicate the sections displayed in Fig. 2. **a**, Western Blots from Figure 2d of total brain homogenate, supernatant, and vessel lysate from the brain of one patient. For the detection of human MFG-E8 and its medin-containing fragments, the membrane was incubated with the anti-human medin antibody (clone 6B3) (*top*). Next, the membrane was stripped, cut into different molecular weight sections, and re-probed with an antibody against α-SMA (clone 1A4, *bottom*). **b**, Western Blots from Fig. 2g of total brain homogenate, RIPA-soluble vascular fraction, and RIPA-insoluble vascular fractions from control patients and AD patients with low/high CAA. Medin-containing MFG-E8 fragments were detected using an anti-human medin antibody (clone 6B3, *top*). Afterwards, the membrane was cut into different molecular weight sections, stripped, and re-probed with antibodies against β-actin (loading control) and Aβ (6e10). Please note: For blots without visible molecular weight markers, these were marked on different photographic films of the same blots (with different exposure times) and transferred through digital overlay.

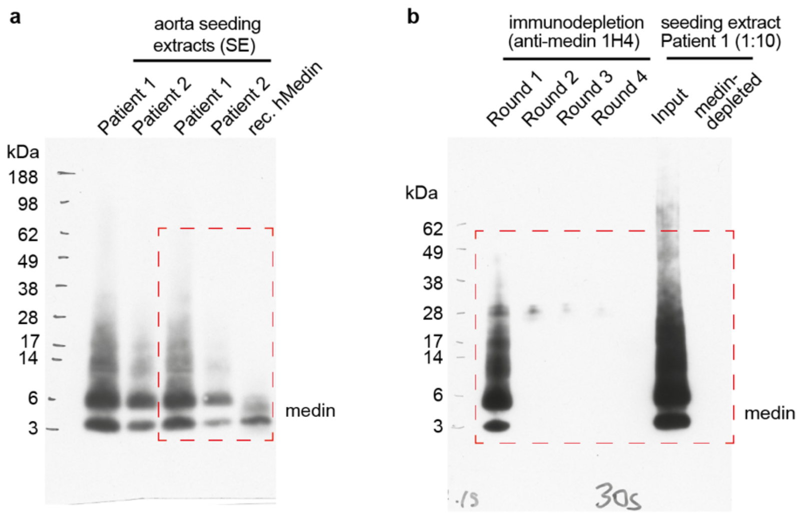

SI Figure 3 | Uncropped Western Blots from main Figure 4.
Red rectangles indicate the sections displayed in Fig. 4. **a**, Western Blot from Fig. 4b for the same volume of aortic seeding extracts of two human AD patients (run in duplicate) as well as recombinant medin as control. Medin-containing proteins were detected using an anti-human medin antibody (clone 6B3). **b**, Western Blot of Fig. 4d showing pulldown of medin from aorta extracts of patient 1 (diluted 1:10) with four rounds of immuno-depletion with anti-medin antibody (clone 1H4). Additionally, seeding extract and immune-depleted material was loaded to confirm medin-depletion using a different anti-human medin antibody (clone 6B3).

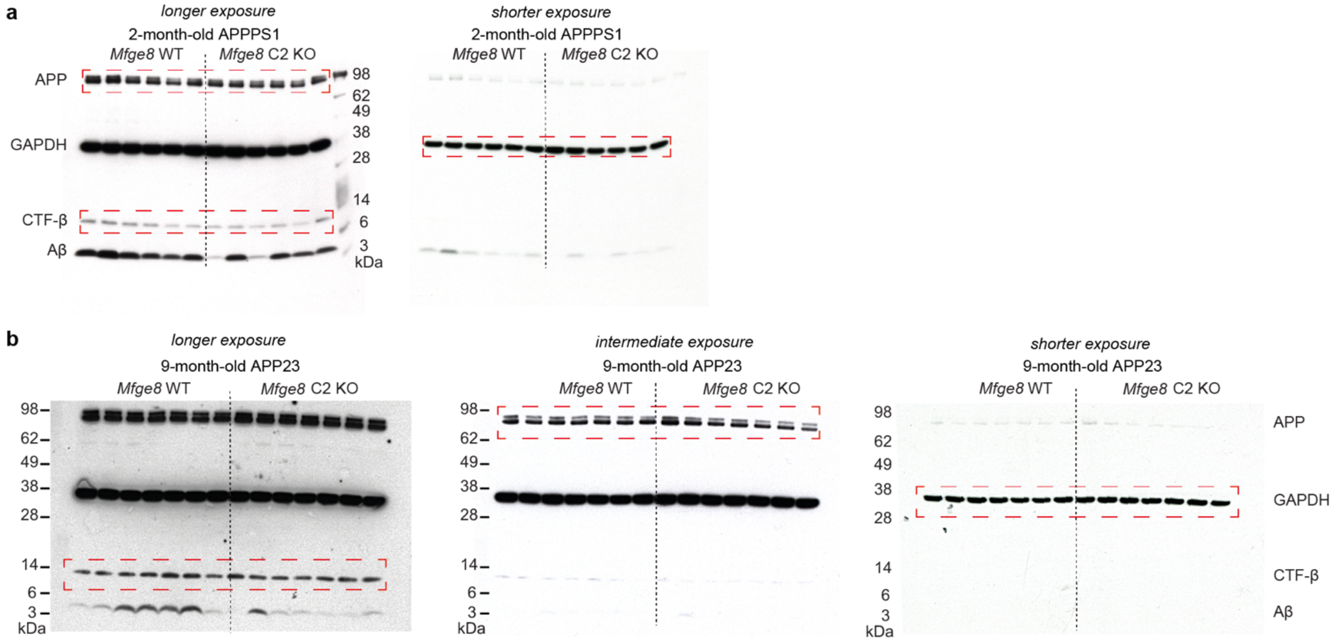

SI Figure 4 | Uncropped Western Blots from Extended Data Figure 2.
Red rectangles indicate the sections displayed in Extended Fig. 2. **a**, Western Blots from Extended Data Fig. 2b of brain homogenates from 2-month-old APPPS1 x *Mfge8* WT/C2 KO animals. APP, CTF-β, and Aβ were detected using the 6e10 antibody with long (*left*) or shorter (*right*) exposure times. GAPDH was used as loading control. **b**, Western Blots from Extended Data Fig. 2d of brain homogenates from 9-month-old APP23 x *Mfge8* WT/C2 KO animals. APP, CTF-β and Aβ were detected using 6e10 antibody at different exposure times. GAPDH was used as loading control. Please note: For blots without visible molecular weight markers, these were marked on different photographic films of the same blots (with different exposure times) and transferred through digital overlay.

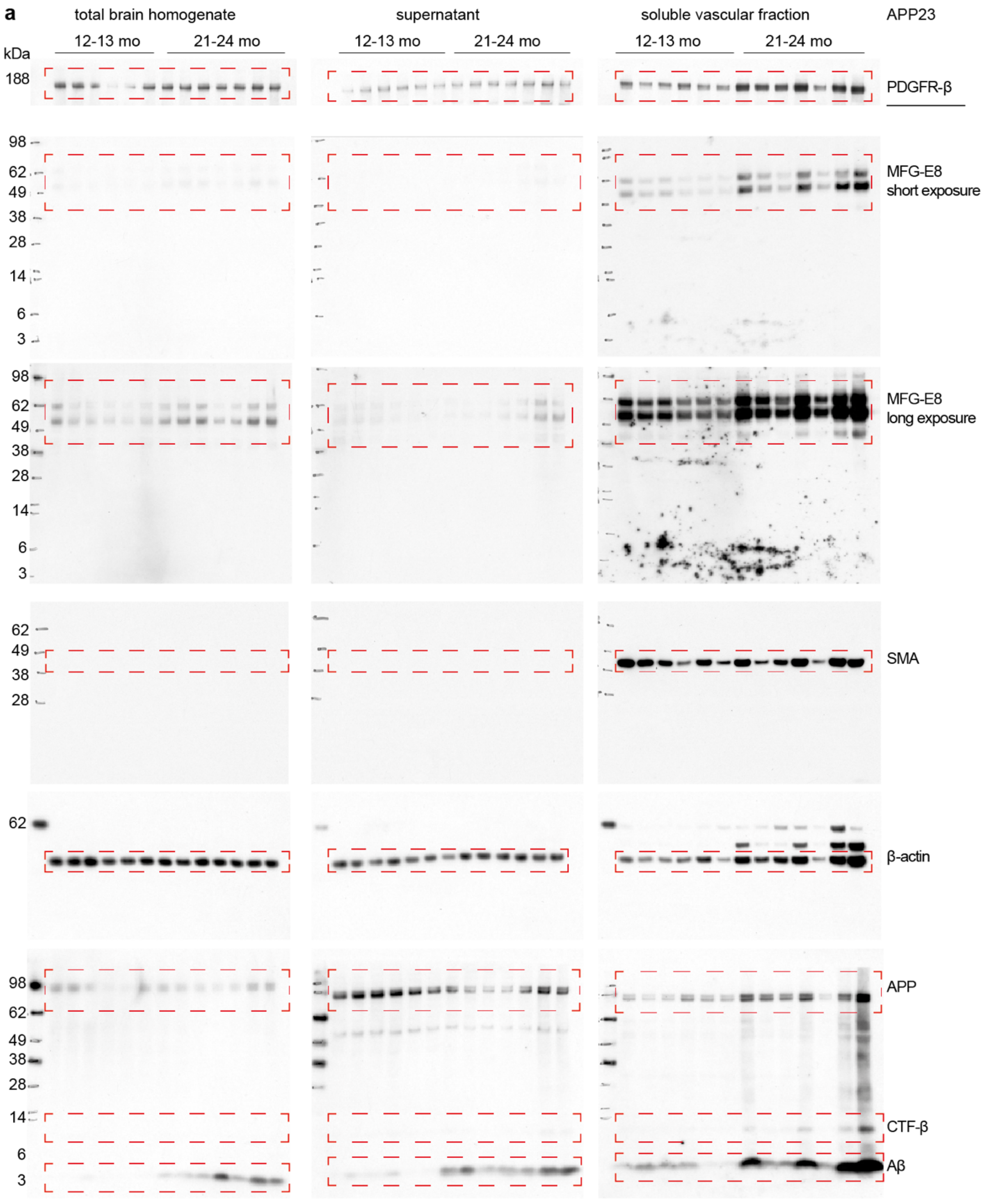

SI Figure 5 | Uncropped Western Blots from Extended Data Figure 5.
Red rectangles indicate the sections displayed in Extended Fig. 5. **a**, Western Blot from Extended Data Fig. 5a of total brain homogenate, supernatant, and the soluble vascular fraction of adult (12-13-month-old) and aged (21-24-month-old) APP23 animals. Following incubation with anti-MFG-E8 and anti-6e10 antibody (detecting APP, CTF-β, Aβ), the membrane was cut into different molecular weight sections (indicated with horizontal black line), stripped and re-probed with antibodies against PDGFR-β and β-actin (loading control). The membrane was stripped again, and re-probed with an α-SMA antibody (clone 1A4). Please note: For blots without visible molecular weight markers, these were marked on different photographic films of the same blots (with different exposure times) and transferred through digital overlay.
