# Supplementary material for: Medin co-aggregates with vascular amyloid-β in Alzheimer’s disease
Source: Nature. 2022 Nov 16;612(7938):123–31. doi: 10.1038/s41586-022-05440-3 (PMC9712113; doi:10.1038/s41586-022-05440-3)
Supplement: Supplementary file 2 — Reporting Summary [file 41586_2022_5440_MOESM2_ESM.pdf]

nature research

Corresponding author(s): Jonas Neher

Last updated by author(s): Sep 28, 2022

Reporting Summary

Nature Research wishes to improve the reproducibility of the work that we publish. This form provides structure for consistency and transparency in reporting. For further information on Nature Research policies, see our Editorial Policies and the Editorial Policy Checklist.

Statistics

For all statistical analyses, confirm that the following items are present in the figure legend, table legend, main text, or Methods section.

n/a	Confirmed	
☐	☒	The exact sample size (*n*) for each experimental group/condition, given as a discrete number and unit of measurement
☐	☒	A statement on whether measurements were taken from distinct samples or whether the same sample was measured repeatedly
☐	☒	The statistical test(s) used AND whether they are one- or two-sided<br>*Only common tests should be described solely by name; describe more complex techniques in the Methods section.*
☐	☒	A description of all covariates tested
☐	☒	A description of any assumptions or corrections, such as tests of normality and adjustment for multiple comparisons
☐	☒	A full description of the statistical parameters including central tendency (e.g. means) or other basic estimates (e.g. regression coefficient) AND variation (e.g. standard deviation) or associated estimates of uncertainty (e.g. confidence intervals)
☐	☒	For null hypothesis testing, the test statistic (e.g. *F*, *t*, *r*) with confidence intervals, effect sizes, degrees of freedom and *P* value noted<br>*Give P values as exact values whenever suitable.*
☒	☐	For Bayesian analysis, information on the choice of priors and Markov chain Monte Carlo settings
☒	☐	For hierarchical and complex designs, identification of the appropriate level for tests and full reporting of outcomes
☒	☐	Estimates of effect sizes (e.g. Cohen's *d*, Pearson's *r*), indicating how they were calculated

Our web collection on statistics for biologists contains articles on many of the points above.

Software and code

Policy information about availability of computer code

Data collection	AxioVision 4.7 software (Zeiss); LSM software 4.2 (Zeiss); ZEN 2012 SP5 (Zeiss); LAS X software (Leica, Leica Application Suite X 3.5.7.23225); G*Power software (v. 3.1.9.6), Sony Software (v. 2.1.5), MACS Quant Analyzer software (MACSQuantify, v2.11).
Data analysis	Stereo Investigator 6 (MBF Bioscience); Fiji (v. 2.3/ 1.53q); Prism 9 software; JMP software (v. 14.2.0 or higher); Imaris 9.7.2; RStudio (v. 1.4.1106); MARS (v. 2.4, BMG Labtech); MSD Discovery Workbench 3.0 (Meso Scale Discovery); Aida (Stella 3200, Raytest).

For manuscripts utilizing custom algorithms or software that are central to the research but not yet described in published literature, software must be made available to editors and reviewers. We strongly encourage code deposition in a community repository (e.g. GitHub). See the Nature Research guidelines for submitting code & software for further information.

Data

Policy information about availability of data

All manuscripts must include a data availability statement. This statement should provide the following information, where applicable:

- Accession codes, unique identifiers, or web links for publicly available datasets
- A list of figures that have associated raw data
- A description of any restrictions on data availability

Source data are provided for Figs. 1d-f/i/j, 2b/e-g, 3b-h, 4/c/e/g, Extended Data Figs 2a-e, 3b/d-f/h, 4a-f, 5b. RNA-sequencing data from human brain were from the ROSMAP study (Mostafavi et al., Nat. Neurosci., 2018) and were provided by the Rush Alzheimer’s Disease Center, Rush University Medical Center, Chicago (https://adknowledgeportal.synapse.org). Protein structures for amyloid fibril assemblies (IDs: 6SHS, 6W0O, 7Q4B, 7Q4M) were obtained from the Protein Data Bank (https://www.rcsb.org/structure/). Reagents and any further information are available on request to the corresponding author.

Field-specific reporting

Please select the one below that is the best fit for your research. If you are not sure, read the appropriate sections before making your selection.

☒ Life sciences ☐ Behavioural & social sciences ☐ Ecological, evolutionary & environmental sciences

For a reference copy of the document with all sections, see nature.com/documents/nr-reporting-summary-flat.pdf

Life sciences study design

All studies must disclose on these points even when the disclosure is negative.

Sample size

A priori power analyses were performed (using G*Power software, version 3.1.9.6) to determine the minimum number of animals for each experiment to achieve a power of 80%. For other experiments, no a priori sample size calculations were performed; rather, sample sizes were chosen based on technical feasibility and/or sample sizes in prior publications of the authors.

Data exclusions

Statistical outliers were identified and removed using the default settings in PRISM 9 software (ROUT method).

Replication

In addition to performing a priori power analyses, we also ran experiments in independent batches of animals to replicate major effects. In vitro experiments for Abeta/medin interaction were replicated 3 times, with 2 technical replicates per run. All other experiments (e.g. Western Blotting, immunostainings) were replicated at least twice. Experiments were replicable in all instances.

Randomization

Animals were randomly allocated into treatment groups, where animals of the same genotype were used, e.g. for i.c. injections. For other experiments, such as comparisons between of transgenic WT/knockout lines or analysis of human tissue samples, randomization was not possible as groups were necessarily pre-defined by genotype or neuropathological assessments, respectively.

Blinding

All stereological quantifications and image analyses for mouse and human tissues were performed by blinded observers. For biochemical analyses and in vitro experiments, observers could not be blinded as samples needed to be pipetted/ordered based on experimental groups.

Reporting for specific materials, systems and methods

We require information from authors about some types of materials, experimental systems and methods used in many studies. Here, indicate whether each material, system or method listed is relevant to your study. If you are not sure if a list item applies to your research, read the appropriate section before selecting a response.

Materials & experimental systems

n/a	Involved in the study	
☐	☒	Antibodies
☒	☐	Eukaryotic cell lines
☒	☐	Palaeontology and archaeology
☐	☒	Animals and other organisms
☐	☒	Human research participants
☒	☐	Clinical data
☒	☐	Dual use research of concern

Methods

n/a	Involved in the study	
☒	☐	ChIP-seq
☐	☒	Flow cytometry
☒	☐	MRI-based neuroimaging

Antibodies

Antibodies used

CD45 A700 (clone 30-F11, BioLegend, catalog number #103128), CD45 FITC (30-F11, Affymetrix Bioscience, #11-0451), CD11b APC (M1/70, BioLegend, #101212), medin (polyclonal, GenicBio Hong Kong, custom-made), Aβ (6E10, BioLegend, #803003), Aβ (CN6 , in house production), murine MFG-E8 (polyclonal goat, R&D systems, AF2805), GAPDH (6C5, Acris Antibodies GmbH, now OriGene Technologies GmbH, # 5G4-6C5), medin (6B3, Prothena, non-commercial), medin (1H4, in house production), Iba1 (polyclonal, WAKO, #019-19741), APP (22C11, Milipore, MAB348), Aldh1l1 (polyclonal, abcam, ab87117), human MFG-E8 (#278918, R&D systems, MAB 27671), SMA (polyclonal, abcam, ab21027, LOT GR3219754-1), SMA (1A4, DAKO, M085129-2), GFAP (polyclonal, Biozol, Z0334), PU.1 (9G7, Cell signaling, #2258), Vimentin (polyclonal, abcam, ab24525), Serpin A3N (polyclonal, R&D systems, AF4709), PDGFR β (28E1, Cell signaling, #3169S), β-actin (polyclonal, abcam, ab8227), Peroxidase-AffiniPure Donkey Anti-Rat IgG (H +L) (polyclonal, Jackson ImmunoResearch Laboratories, 712-035-153), Peroxidase-AffiniPure Bovine Anti-Goat IgG (H+L) (polyclonal, Jackson ImmunoResearch Laboratories, 805-035-180), Peroxidase-AffiniPure Donkey Anti-Rabbit IgG (H+L) (polyclonal, Jackson ImmunoResearch Laboratories, 711-035-152), Peroxidase AffiniPure Goat Anti-Mouse IgG + IgM (H+L) (polyclonal, Jackson ImmunoResearch Laboratories, 115-035-068).

Validation

Individual antibodies with details are described in Materials and Methods. All commercial antibodies used in this study were selected based on provided manufacturer's validation data or reference publications; validation of in-house antibodies was performed in previous or the current studies of the authors:
CD11b and CD45 (Wendeln et al., 2018), medin (GenicBio Hong Kong, Migrino et al., 2017), Aβ (6E10, BioLegend, #803003), Aβ (CN6 , Eisele et al., 2010), murine MFG-E8 (Degenhardt et al., 2020), GAPDH (6C5, Acris Antibodies GmbH), medin (6B3, Fig.4, validated by

medin-depletion by anti-medin clone 1H4), medin (1H4, Degenhardt et al., 2020), Iba1 (Varvel et al., 2015), APP (22C11, MAB348), Aldh1l1 (Beyer et al., 2021), human MFG-E8 (Jinushi et al., 2008), SMA (ab21027, Merlini et al., 2016), SMA (1A4, DAKO, M085129-2), GFAP (Biozol, Z0334), PU.1 (Ueki et al., 2008), Vimentin (ab24525, Habib et al., 2020), Serpin A3N (Habib et al., 2020), PDGFR-β (28E1, Cell signaling, #3169S), β-actin (polyclonal, abcam, ab8227), Peroxidase-AffiniPure Donkey Anti-Rat IgG (H+L) (polyclonal, Jackson ImmunoResearch Laboratories, 712-035-153), Peroxidase-AffiniPure Bovine Anti-Goat IgG (H+L) (polyclonal, Jackson ImmunoResearch Laboratories, 805-035-180), Peroxidase-AffiniPure Donkey Anti-Rabbit IgG (H+L) (polyclonal, Jackson ImmunoResearch Laboratories, 711-035-152), Peroxidase AffiniPure Goat Anti-Mouse IgG + IgM (H+L) (polyclonal, Jackson ImmunoResearch Laboratories, 115-035-068).

Animals and other organisms

Policy information about studies involving animals; ARRIVE guidelines recommended for reporting animal research

Laboratory animals	Male and female mice, from 2-24 month-old, of the following lines were used: C57BL/6J and Mfge8 C2 knockout (C57BL/6J-Mfge8 Gt(KST227)Byg) mice; APP transgenic mouse lines: APPDutch C57BL/6J-Tg(Thy1-APPDutch), APPPS1 (C57BL/6J-Tg(Thy1-APPK670N;M671L and Thy1-PS1L166P), APP23 (C57BL/6J-Tg(Thy1-APPK670N;M671L) and C57BL/6 JNpa-Tg(Thy1App)23/1Sdz (APP23N); sex and animal numbers/ages are reported in the figure legends for individual experiments. Animals were kept under specific pathogen-free conditions, with ad libitum food and water, a light cycle from 7am-7pm, a temperature of 22±2ºC and a humidity level of 55±10%.
Wild animals	No wild animals were used in this study.
Field-collected samples	No field-collected samples were used in this study.
Ethics oversight	All experiments were performed in accordance with German veterinary office regulations (Baden-Württemberg) and were approved by the local authorities for animal experimentation (Regierungspräsidium) of Tübingen, Germany. A statement to this effect is included in the manuscript.

Note that full information on the approval of the study protocol must also be provided in the manuscript.

Human research participants

Policy information about studies involving human research participants

Population characteristics	Details for human tissue donor characteristics are provided in Supplementary Information Tables 1-2.
Recruitment	Patients were recruited by the tissue banks.
Ethics oversight	This study was ethically approved by Liverpool Bio-Innovation Hub (project approval reference 15-06 and 18-07). The LBIH Biobank confers ethical approval for the use of samples through their ethical approval as a Research Tissue Bank (REC reference 14/NW/1212, NRES Committee North West–Haydock). Human brain tissue (Extended Data Table 1) was obtained from the Queen Square Brain Bank for Neurological Disorders (UCL Institute of Neurology, London, UK; approval protocol No: EXTMTA5/16) and the Emory University Alzheimer's Disease Research Center (IRB 00045782) with informed consent obtained from all participants or their families. This study was also approved by the ethical committee of the Medical Faculty, University of Tübingen, Germany (Protocols: 354/2016BO2, 832/2021BO2, 369/2021BO2).

Note that full information on the approval of the study protocol must also be provided in the manuscript.

Flow Cytometry

Plots

Confirm that:

☒ The axis labels state the marker and fluorochrome used (e.g. CD4-FITC).

☒ The axis scales are clearly visible. Include numbers along axes only for bottom left plot of group (a 'group' is an analysis of identical markers).

☒ All plots are contour plots with outliers or pseudocolor plots.

☒ A numerical value for number of cells or percentage (with statistics) is provided.

Methodology

Sample preparation	One day prior to microglial isolation, mice were intraperitoneally injected with 17.5 µl per g bodyweight of the amyloid dye Methoxy-X04 (4% vol of 10 mg/ml Methoxy-X04 in DMSO, 7.7% vol CremophoreEL in 88.3% vol PBS). Microglia were isolated as previously described4,5. Briefly, the neocortex was dissected and minced in ice-cold Hanks Buffered Salt Solution (HBSS, 15 mM HEPES, 0.54% D-Glucose, 0.1% DNase (w/v)). The minced tissue was sequentially homogenized in glass Dounce and Potter homogenizers (Wheaton). Tissue suspension was filtered through a 70 µm cell strainer (BD Biosciences) and centrifugated at 300 g for 15 min at 4°C in a swinging-bucket rotor. The pellet was resuspended in 70% Percoll solution (Healthcare) and centrifuged for 30 min at 800 g at 4°C through a 70%, 37% and 30% isotonic Percoll gradient. Cells were recovered from the 70/37% interphase and washed with fluorescence-activated cell sorting (FACS) buffer (1xHBSS, 2% FCS, 10 mM EDTA) by centrifugation at 300 g for 15 min at 4°C. For blocking of non-specific Fc receptor-mediated antibody binding, the cell pellet was resuspended in FACS buffer, and Fc-block (BD, 1:400) was added for 10 min. Cells were stained

	with anti-mouse CD45 A700 (Biolegend, 1:200) or anti-mouse CD45 FITC (Affymetrix Bioscience, 1:100) and anti-CD11b APC (Biolegend, 1:200) for 15 min at 4°C. After washing, the pellet was resuspended in FACS buffer containing 25 mM HEPES. CD11bhigh/CD45low-positive microglial cells were sorted with a Sony SH800 flow cytometer in FACS buffer containing 25 mM HEPES.
Instrument	Sony SH800Z flow cytometer or MACSQuant Analyzer.
Software	Sony Software version 2.1.5 or MACS Quant Analyzer software (MACSQuantify, v2.11).
Cell population abundance	Cell purity after sorting is >99% CD45int/CD11bhigh as determined during optimisation procedures for the protocol described, using re-sorting of the purified population.
Gating strategy	Gating strategy is shown in Extended Data Fig.4a, where the cell population was generously gated in FSC/SSC based on a density plot. Single cells were then identified based on FSC-A vs. FSC-W (not shown). Microglia were identified on CD45intermediate/CD11bhigh signal. In this microglial population, Methoxy-X04 positive cells were identified, with gating based on signal from wildtype cells. The microglial cells were analysed by MACS Quant Analyzer and sorted by Sony SH800Z flow cytometer.

☒ Tick this box to confirm that a figure exemplifying the gating strategy is provided in the Supplementary Information.
